# Leveraging coevolutionary insights and AI-based structural modeling to unravel receptor–peptide ligand-binding mechanisms

**DOI:** 10.1073/pnas.2400862121

**Published:** 2024-08-06

**Authors:** Simon Snoeck, Hyun Kyung Lee, Marc W. Schmid, Kyle W. Bender, Matthias J. Neeracher, Alvaro D. Fernández-Fernández, Julia Santiago, Cyril Zipfel

**Affiliations:** ^a^Department of Plant and Microbial Biology (IPMB), Zurich-Basel Plant Science Center, University of Zurich, Zurich 8008, Switzerland; ^b^The Plant Signaling Mechanisms Laboratory, Department of Plant Molecular Biology, University of Lausanne, Lausanne 1015, Switzerland; ^c^MWSchmid GmbH, Glarus 8750, Switzerland; ^d^The Sainsbury Laboratory, University of East Anglia, Norwich Research Park, Norwich NR4 7UH, United Kingdom

**Keywords:** receptor, ligand, peptide, structure prediction, evolution

## Abstract

Secreted signaling peptides are central regulators of growth, development, and stress responses, but specific steps in the evolution of these peptides and their receptors are not well understood. Also, the molecular mechanisms of peptide–receptor binding are only known for a few examples, primarily owing to the limited availability of protein structural determination capabilities to few laboratories worldwide. Plants have evolved a multitude of secreted signaling peptides and corresponding transmembrane receptors. Stress-responsive SERINE RICH ENDOGENOUS PEPTIDES (SCOOPs) were recently identified. Bioactive SCOOPs are proteolytically processed by subtilases and are perceived by the leucine-rich repeat receptor kinase MALE DISCOVERER 1-INTERACTING RECEPTOR-LIKE KINASE 2 (MIK2) in the model plant *Arabidopsis thaliana*. How SCOOPs and MIK2 have (co)evolved, and how SCOOPs bind to MIK2 are unknown. Using in silico analysis of 350 plant genomes and subsequent functional testing, we revealed the conservation of MIK2 as SCOOP receptor within the plant order Brassicales. We then leveraged AI-based structural modeling and comparative genomics to identify two conserved putative SCOOP–MIK2 binding pockets across Brassicales MIK2 homologues predicted to interact with the “SxS” motif of otherwise sequence-divergent SCOOPs. Mutagenesis of both predicted binding pockets compromised SCOOP binding to MIK2, SCOOP-induced complex formation between MIK2 and its coreceptor BRASSINOSTEROID INSENSITIVE 1-ASSOCIATED KINASE 1, and SCOOP-induced reactive oxygen species production, thus, confirming our in silico predictions. Collectively, in addition to revealing the elusive SCOOP–MIK2 binding mechanism, our analytic pipeline combining phylogenomics, AI-based structural predictions, and experimental biochemical and physiological validation provides a blueprint for the elucidation of peptide ligand–receptor perception mechanisms.

## Significance

This study presents proof-of-concept for a rapid and inexpensive alternative to classical structure-based approaches for resolving ligand–receptor binding mechanisms. It relies on a multilayered bioinformatic approach that leverages genomic data across diverse species in combination with AI-based structural modeling to identify true ligand and receptor homologues and subsequently predict their binding mechanisms. In silico findings were validated by multiple experimental approaches, which investigated the effect of amino acid changes in the proposed binding pockets on ligand-binding, complex formation with a coreceptor essential for downstream signaling, and activation of downstream signaling. Our analysis combining evolutionary insights, in silico modeling, and functional validation provides a framework for structure–function analysis of other peptide–receptor pairs, which could be easily implemented by most laboratories.

Secreted signaling peptides are central regulators of growth, development, and stress responses in Eukaryotes. Plants, in particular, have evolved hundreds to thousands of such peptides and corresponding transmembrane receptors to regulate their growth and development in the face of an ever-changing environment (1, 2). Knowledge of the exact binding mechanisms of these peptides to their transmembrane receptors is however limited to a handful of examples (3–11), mostly owing to the limited capability of most laboratories to perform structural determination of ligand–receptor complexes. This “classical” approach also faces technical challenges such as obtaining sufficient amounts of protein for crystallization (crystallography) or cryogenic electron microscopy (cryo-EM) experiments, getting well-diffracting crystals, and complex size (cryo-EM) and in general is limited to perform high-throughput structural determination studies (12). Moreover, time, effort, and cost of traditional crystal structure as well as cryo-EM determination approaches are a major constraint (13). Therefore, alternative approaches are needed to accelerate the study of interactions at the receptor–ligand interface.

The family of stress-responsive SERINE RICH ENDOGENOUS PEPTIDES (SCOOPs) was identified in 2019 in the model plant *Arabidopsis thaliana* (hereafter, *Arabidopsis*) (14). Minimal bioactive SCOOPs are 13 to 15 amino acid (AA) peptides proteolytically processed by subtilases from PROSCOOP precursors (14–17). Most *Arabidopsis* SCOOPs harbor the conserved “SxS” motif that is essential for the bioactivity of the best characterized SCOOP, SCOOP12 (14, 18). Recently, a comprehensive annotation of *PROSCOOP* genes in the *Arabidopsis* Col-0 genome revealed the existence of 50 putative SCOOP peptides, making the SCOOPs one of the largest families of signaling peptides identified in flowering plants so far (17).

Plants employ germline-encoded receptor kinases (RKs) and receptor proteins (RPs) to sense their extracellular environment and coordinate their growth and development in response to endogenous and exogenous cues (19). The most common ectodomain is a series of leucine-rich repeats (LRRs), which mediate ligand-binding and coreceptor association (20–23). SCOOPs were recently identified to be perceived by the LRR-RK MALE DISCOVERER 1-INTERACTING RECEPTOR-LIKE KINASE 2 (MIK2) (15, 18). SCOOPs induce the complex formation between MIK2 and the common LRR-RK coreceptor BRASSINOSTEROID INSENSITIVE 1-ASSOCIATED KINASE 1 (BAK1) (15, 18). Notably, MIK2 and SCOOPs have been since implicated in multiple aspects of plant growth, development, and response to both biotic and abiotic stresses, therefore highlighting their biological relevance (14–18, 24–29). Despite these advances, the evolutionary history of SCOOPs and MIK2, and how SCOOPs are perceived by MIK2 remain mostly unclear. The latter is particularly intriguing given that SCOOPs have divergent sequences apart from the conserved SxS motif, and their sequences also suggest a different mode of binding compared to that of other characterized plant peptide–receptor pairs (20, 30, 31).

Multiple studies recently leveraged (pan)genomic data across and beyond plant families to gain insights into the structure–function mechanisms of RKs and RPs (32–36). Besides comparative genomics, protein structural modeling is now widely accessible. AlphaFold-Multimer (AFM) is an extension of AlphaFold2 (AF2) developed by DeepMind (37). Whereas AF2 predicts individual protein structures, AFM predicts structures of protein complexes with relatively high accuracy for ~23% of heteromeric interfaces (37). Although AF2 was only trained on monomer chains, it was quickly realized—owing to the idea that the molecular interactions governing protein folding are also of importance for protein–protein docking—that AF2 could also predict protein–protein models. Subsequently, AFM was released, an extension of AF2 specifically trained to predict protein complex structures with increased accuracy (12, 37, 38). Multiple in silico studies quickly reported AFM suitability for predicting peptide–protein interactions by challenging it against known interactors. AFM outperforms state-of-the-art peptide–protein complex modeling (37, 39–44). Moreover, a new and improved AlphaFold model was recently released, AlphaFold 3 (AF3) (45).

Here, we pioneer and functionally validate a relatively quick approach, combining the use of AFM/AF3 and comparative genomics, to predict the ligand-binding pockets of an LRR-RK. Two binding pockets on MIK2 were predicted to interact with the conserved SxS motif of the otherwise sequence-divergent SCOOPs identified within the plant order Brassicales. The AI predictions were supported by strong conservation of the predicted binding sites in validated MIK2 homologues across Brassicales. Site-directed mutagenesis of these binding pockets impaired SCOOP12-binding, SCOOP12-induced MIK2–BAK1 complex formation, and reactive oxygen species (ROS) production triggered by a multitude of SCOOPs.

## Results

### PROSCOOPs Are Exclusive to the Plant Order Brassicales, Show Diverse Conservation Patterns, and Most Harbor the SxS Motif.

**Fig. 1.**
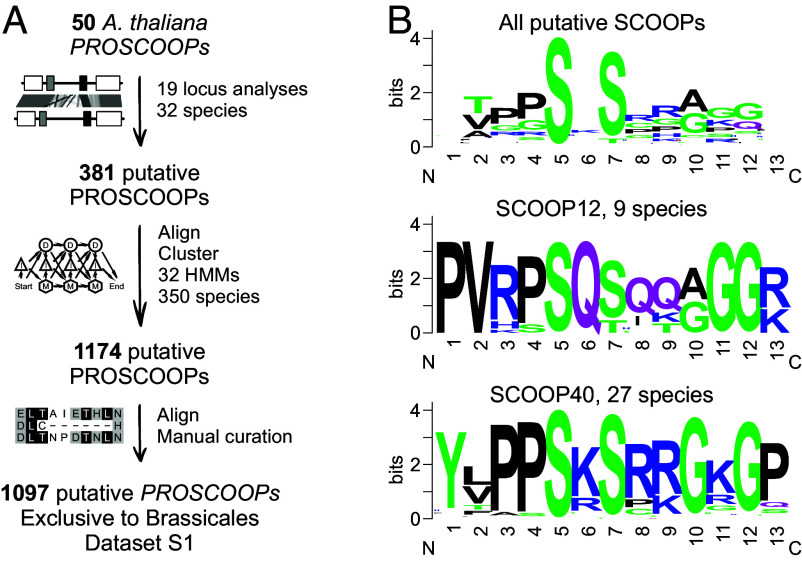
In silico mining of putative PROSCOOPs reveals that the majority harbors the SxS motif, all PROSCOOPs are exclusively identified within the Brassicales. (*A*) Schematic of the bioinformatic pipeline; a locus analysis facilitated PROSCOOP HMM-based mining across the plant kingdom, PROSCOOP candidates were subsequently manually curated. (*B*) Sequence motif analysis of respectively all identified putative SCOOPs, SCOOP12, and SCOOP40. Sequence logos were generated using Dataset S1 and WebLogo server (https://weblogo.berkeley.edu/logo.cgi).

To shed light on PROSCOOP emergence and SCOOP conservation, a locus analysis was performed across 32 Brassicales species for each of the 19 *Arabidopsis* *PROSCOOP* loci that harbor the 50 *Arabidopsis* *PROSCOOPs* earlier identified (14–17). This strategy leveraged synteny and increased the number of putative *PROSCOOP*s to 381, facilitating a cluster analysis and the creation of 32 hidden Markov models (HMMs). The HMM profiles were then used as a query for an hmmsearch against 350 predicted proteomes across the entire plant kingdom and some unicellular algae (46). Manual curation of the resulting dataset settled on a total of 1,097 putative *PROSCOOPs* identified in 32 species (Fig. 1*A* and Dataset S1).

*Cleome violacea* is the earliest divergent species in which putative *PROSCOOP*s were identified. Three of them reside in the same clusters as *Arabidopsis* *PROSCOOP40* and *48-49* (Dataset S1). Therefore, putative *PROSCOOP*s were exclusively found within Brassicales species, which diverged ~39 Mya (47). Five out of 32 maintained *PROSCOOP* clusters do not contain any *Arabidopsis* *PROSCOOP*s, suggesting that our bioinformatic pipeline was able to identify novel SCOOPs besides *Arabidopsis* SCOOP-homologues across species. Like the SCOOPs identified in *Arabidopsis*, Brassicales SCOOP sequences are divergent aside from the characterized SxS motif, and a minority harbor an “SxT” motif instead (Fig. 1*B*). Threonine (T), like serine (S), has a polar uncharged side chain and the ability to form hydrogen bonds. In addition, T also has a methyl group that allows the AA to establish van der Waals contacts with other nonpolar groups. In contrast to SCOOPs in general, motif analysis of SCOOP clusters reveals strong conservation of certain 13mer SCOOP sequences, suggestive of conserved function for some SCOOPs across species (Fig. 1*B*).

Although our search is biased by the annotation quality of the available genome assemblies (2), it allows us to acquire a general understanding of the sequence conservation of (PRO)SCOOPs within and across species. For example, clusters containing *PROSCOOP13* and *16*, *37-39*, and *40* are represented in at least 27 out of 32 Brassicales proteomes, indicating a strong conservation after initial appearance during the evolution of Brassicaceae. In contrast, *PROSCOOP5*, *34,* and *43* sequences seem to be relatively unique as they did not cluster with any other sequences after the initial locus analysis within 32 species. *PROSCOOP29, 30, 42, 44, 45, 46,* and *47* clustered with just one other sequence. *PROSCOOP2*, *7*, *12,* and *14* are found in less than 10 out of 32 Brassicales species. Independent of their evolutionary conservation within the Brassicales, 13mer SCOOP sequences can be strongly conserved (Dataset S1).

To test the conservation of plant responses to SCOOPs, we measured the production of ROS—a hallmark of LRR-RK activation—triggered by SCOOPs. SCOOP12 was used as it is the best characterized SCOOP (14, 15, 25, 26). Moreover, *Arabidopsis* SCOOP12 was earlier shown to induce apoplastic ROS production in *Brassica napus* (14), even though our dataset indicates that *B. napus* does not have a bona fide *PROSCOOP12*-homologue in the cluster containing *Arabidopsis* PROSCOOP12. Moreover, relative to SCOOP12, the maximum AA similarity with any predicted *B. napus* SCOOP (13 AA) is ~54% (SCOOP27 cluster, Dataset S1). Besides SCOOP12, 13, 16, and 24 were selected for testing, based on the following three criteria: A) relatively conserved across the Brassicales, B) strong sequence conservation of the predicted active SCOOP, and C) induction of robust ROS production upon SCOOP treatment in *Arabidopsis* (17). Brassicaceae plants were carefully selected to cover the diversity of the plant family (*SI Appendix*, Fig. S1). *C. violacea* was selected as a close outgroup for the family of the Brassicaceae (order of the Brassicales). We measured ROS production following application of the selected SCOOPs in these plants and included the immune elicitor flg22 as a positive control to test the suitability of our assay for each species. *Carica papaya*, a species from a relatively close outgroup for the Brassicales, did not respond to flg22 in our ROS production assay and was therefore excluded from further analyses. Besides *Arabidopsis*, *Brassica rapa, Eutrema salsugineum, Euclidum syriacum, Diptychocarpus strictus,* and *C. violacea* responded significantly to at least two out of four tested SCOOPs (*SI Appendix*, Fig. S2). Hence, phylogenetic and experimental evidence suggests that SCOOP perception occurs within the Brassicales.

### MIK2 Is a Brassicales-Specific, Conserved SCOOP Receptor.

**Fig. 2.**
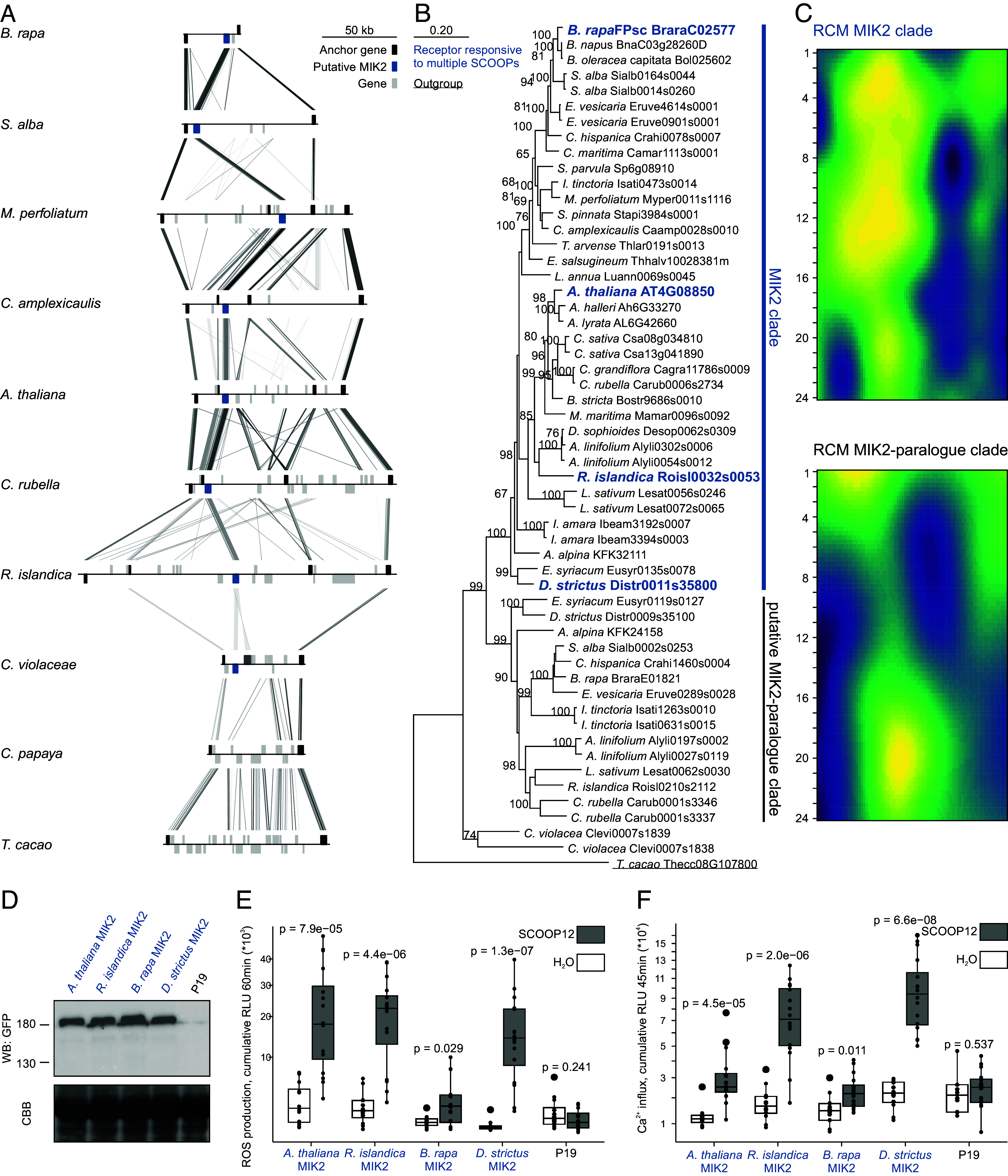
MIK2 is specific to and strongly conserved within the Brassicales family. (*A*) Anchor, putative *MIK2*, and other genes are colored as per legend. Locus comparison of eight contiguous MIK2 loci within the Brassicales and the relatively close outgroups *C. papaya* and *T. cacao*. Blast hits between loci are indicated with lines (e-value <1e-06) with score according to grayscale gradient and darker grays indicating higher similarity. Gene orientation is depicted by the position of them above or below the line. (*B*) A phylogenetic analysis of putative MIK2 homologues and paralogues. Maximum-likelihood analysis bootstrap values are indicated, and only values higher than 65 are shown. The scale bar represents 0.2 AA substitutions per site. An LRR-RK that resides at the conserved MIK2 locus of *T. cacao* was used as an outgroup to root the phylogenetic gene tree and is underlined. The functionally validated MIK2 receptors of *A. thaliana, R. islandica, B. rapa,* and *D. strictus* are highlighted in blue and bold as they confer SCOOP-induced ROS production and increase in cytosolic Ca^2+^ concentration upon heterologous expression in *Nicotiana benthamiana*, as shown respectively in panel *E* and *F* and *SI Appendix*, Fig. S3. These four validated MIK2s fall within the labeled “MIK2 clade.” (*C*) Repeat conservation mapping (RCM) of the putative MIK2 (n = 37) and potential MIK2 paralogues (n = 15) clade. Each row represents the solvent-exposed AAs of a single repeat of the LRR. The color represents the center-weighted regional conservation score for the 5 × 5 set of boxes that centers on that box; yellow indicates the most conserved regions and blue indicates the most divergent regions. (*D*) Western blot of the heterologously expressed MIK2 and MIK2 homologues in *N. benthamiana*. Tissue was harvested 48 h after construct infiltration in *N. benthamiana*. The western blot was probed with α-GFP (B-2) Horseradish peroxidase (HRP) as the receptor had a C-terminal GFP tag (*Top*) and subsequently stained with CBB as a loading control (*Bottom*). See Dataset S6 for original tiff files. (*E* and *F*) Shown are ROS production (*E*) and increase in Ca^2+^ cytosolic concentrations (*F*), respectively 4 to 60 min and 3 to 45 min, in cumulative relative luminescence units (RLUs) post treatment with H_2_O (white) or the peptide SCOOP12 (1 μM, gray). Four independent biological replicates (n = 4 plants) were performed, with each biological replicate represented by four technical replicates. Significance was tested by performing a paired Wilcoxon rank-sum test.

To explore the gain of SCOOP response in relation to its defined receptor in *Arabidopsis*, putative MIK2 homologues were identified in silico following a multilayered approach similar to the (PRO)SCOOP mining. Initially, leveraging synteny, a locus analysis identified 38 putative MIK2s within 32 Brassicales species. The advantage of locus analysis against genome-wide searches is the probable common evolutionary origin of the gene of interest. Subsequently, an HMM profile was created and used to interrogate 350 species across the plant kingdom (46). In this way, we also included putative MIK2 homologues and putative MIK2 paralogues RKs within and outside the Brassicales, which do not necessarily reside within the conserved *MIK2* locus. Subsequently, a phylogenetic analysis was performed and a clade containing all initial putative MIK2s was extracted. After manual curation of the alignment (24 LRRs, no gaps in other conserved domains), 17 novel LRR-RKs were identified besides the earlier identified 38. As a relatively close outgroup of the Brassicales, we also investigated the *MIK2* locus of *Carica papaya* and found that the *MIK2* locus of *C. papaya* does not contain any LRR-RK-encoding gene (Fig. 2*A*). In contrast, earlier diverged species such as *Theobroma cacao* do harbor one or more LRR-RK-encoding genes at the *MIK2* locus, but with a relatively low sequence similarity to *Arabidopsis* MIK2 (Thecc08G107800: 61%). In summary, no putative MIK2 homologues were identified outside the Brassicales.

Two maximum-likelihood phylogenetic analyses—using either sequences of the extracellular domain only or the full protein—were performed with the 54 putative MIK2s. Thecc08G107800 (from *T. cacao*) was included in the analysis to root the tree (Fig. 2*B*). Both strategies identified a putative MIK2 clade containing 37 putative Brassicaceae MIK2 homologues, all residing at the *MIK2* loci, with an AA similarity of 86 to 97% relative to *Arabidopsis* MIK2. A common function of all LRR-RKs within the putative MIK2 clade is likely as a Repeat Conservation Mapping (RCM) analysis (48) identified conserved sites in a putative ligand-binding region spanning LRR1-15 on the predicted surface of the LRR ectodomain (Fig. 2*C*) (34).

In contrast, 16 out of 17 novel MIK2 candidates cluster in a distinct clade, show a relatively low conservation in the RCM between LRR1-15 (Fig. 2*C*), and none of these LRR-RK-encoding genes reside at one of the earlier identified *MIK2* loci. All members within this clade, hereafter described as the putative MIK2-paralogue clade also have a relatively lower AA similarity with *Arabidopsis* MIK2 of 74 to 81%. The last novel MIK2 candidate, Clevi0007s1838, resides at the *MIK2* locus of *C. violacea* (Brassicales) and clusters together with its neighboring gene *Clevi0007s1839* but separate from all other putative MIK2 homologues and -paralogues within the Brassicaceae. Clevi0007s1839 shares approximately 79% AA sequence similarity with *Arabidopsis* MIK2.

Next, we investigated the function and relationship of individual putative MIK2 homologues within the putative MIK2 clade. Besides *Arabidopsis* *MIK2*, three genes—*Roisl0032s0053* (from *Rorippa islandica*), *BraraC02577* (from *B. rapa*), and *Distr0011s35800* (from *D. strictus*)—were selected as representatives for the major subdivisions within the Brassicaceae, cloned, and transiently expressed in the non-Brassicaceae model species *Nicotiana benthamiana*, which is insensitive to SCOOPs (14, 18). As in previous experiments testing Brassicales species for SCOOP responsiveness, we initially used SCOOP12 as it is the best characterized SCOOP to validate the function of our putative MIK2s and the putative MIK2 clade in general. Receptor function was measured using SCOOP-induced ROS production and increase in cytosolic Ca^2+^ concentration. Transient expression of all three representative genes conferred SCOOP12-induced activation of both tested hallmarks of LRR-RK signaling, consistent with SCOOP recognition enabled by the MIK2 clade (Fig. 2 *E* and *F*). Similar to our analysis of SCOOP perception across species, we then tested whether transient expression of the three MIK2 homologues could confer response to additional SCOOPs, namely SCOOP13, 16, and 24. *Arabidopsis* SCOOP13 and 16 differ by two AAs, are part of the same cluster that was found in 28 species and have a strong sequence conservation across them. Comparable ROS bursts were observed for both SCOOPs as all MIK2 homologues conferred responsiveness except *B. rapa* MIK2 (BraraC02577) (*SI Appendix*, Fig. S3). Similarly, transient expression of *B. rapa* MIK2 did not lead to significant ROS production (Wilcoxon-ranked sum, *P* = 0.14) upon treatment with SCOOP24, a strongly sequence-conserved SCOOP found in 22 Brassicaceae (Dataset S1). Nevertheless, all tested SCOOPs did result in an increase of cytosolic Ca^2+^ concentration, often a more sensitive marker for RK signaling relative to ROS production. In summary, SCOOP perception across the Brassicales is correlated with the in vivo function of MIK2 homologues.

### The Conserved SCOOP Motif SxS Is Predicted to Interact with Two MIK2 Binding Pockets.

**Fig. 3.**
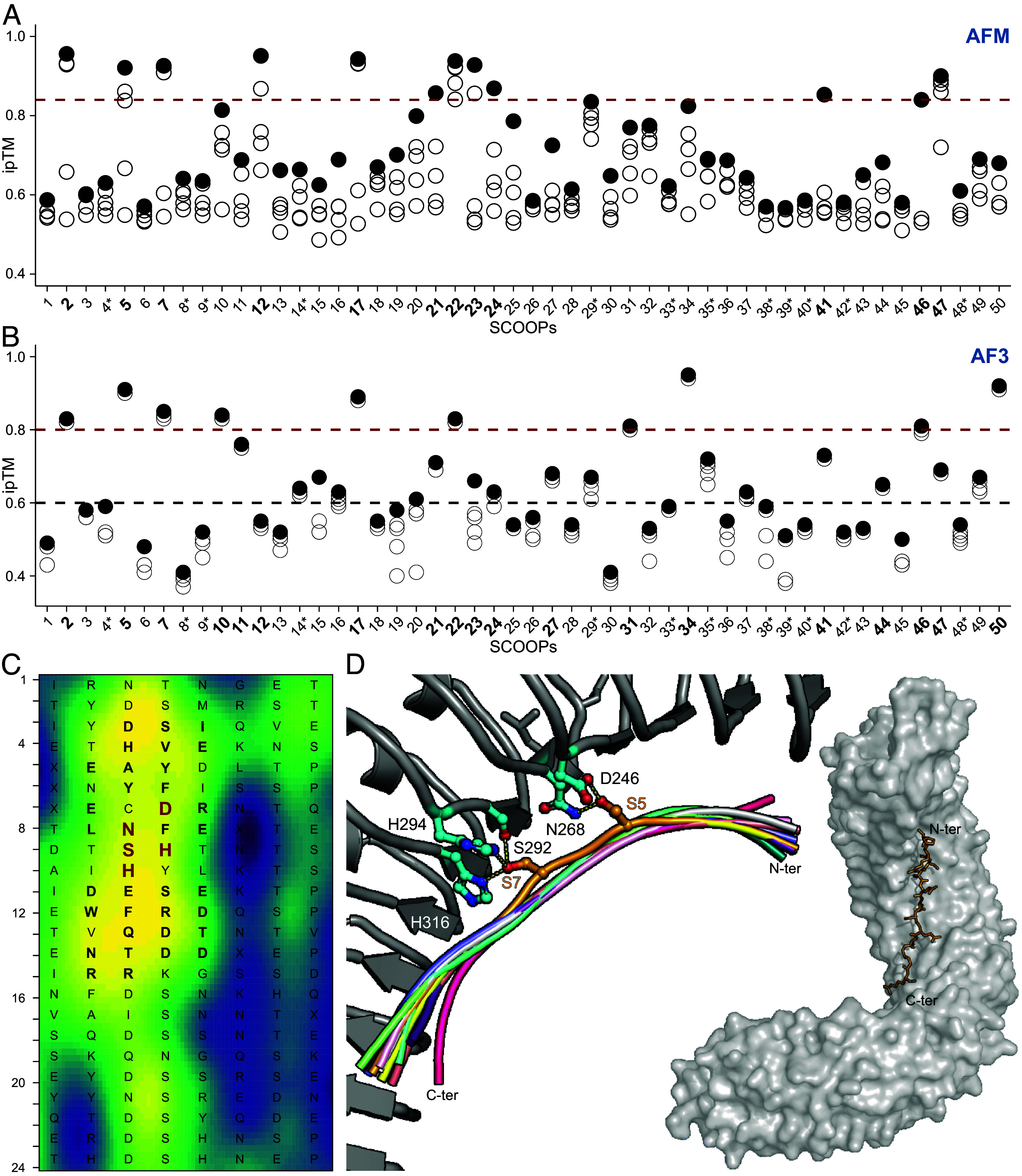
AFM and AF3 predict that the SxS motif interacts with two conserved binding pockets of MIK2. (*A* and *B*) AFM and AF3 predict a high ipTM scores for multiple *Arabidopsis* SCOOPs in complex with the MIK2 receptor. Highlighted in bold are consistent predicted interactions between the SCOOPs SxS motif and MIK2. Considering AFM, these are the 12 highest scoring (ipTM > 0.84, red dotted line). The AF3 guidelines, as reflected by the ipTMs of 19 of our predictions, state that ipTM values higher than 0.8 represent confident high-quality predictions. ipTM values between 0.6 and 0.8 are within a gray zone where predictions could be correct or incorrect. The AF3 cutoffs are depicted with a red and black dotted line. Asterisks indicate the use of the 15mer instead of 13mer SCOOP for complex prediction. (*C*) RCM of the putative MIK2 (n = 37) clade. Each row represents a single repeat of the LRR, with each colored box representing a solvent-exposed AA position. The consensus sequence of the 37 MIK2s is depicted, single AAs are enlarged and in bold in case an interaction with SCOOP12 was predicted by AFM, and additionally highlighted in red in case AFM predicts an interaction with the conserved SxS motif of SCOOPs. The color represents the center-weighted regional conservation score for the 5 × 5 set of boxes that centers on that box; yellow indicates the most conserved regions and blue indicates the most divergent regions. (*D*) Structural superposition of 11 SCOOPS in cartoon representation (2, 5, 7, 12, 17, 21–24, 45, 46) with the two conserved S (depicted in orange sticks) anchoring the peptide to the receptor binding canyon. S5 mediates hydrogen bond interactions with the conserved residues D246 and N268 (depicted in cyan sticks). S7 conserved binding pocket is composed by S292, H316, and H294 (depicted in cyan sticks). On the *Right* side, an AFM model prediction of MIK2 in complex with SCOOP12 is depicted. SCOOP12 is predicted to bind to the MIK2 internal binding groove in a fully extended conformation. MIK2 is depicted in gray surface and SCOOP12 in yellow sticks.

To understand how MIK2 perceives SCOOP ligands, we used AFM in combination with RCM. Because peptide ligands are perceived by LRR ectodomains, and since AlphaFold does not correctly orient ecto- and cytosolic domains with respect to transmembrane domains (49), we elected to predict the interaction between SCOOPs and the isolated MIK2 ectodomain. Initially, we did not include the coreceptor BAK1 as it only gets recruited in the SCOOP12–MIK2 complex upon ligand-binding (15, 18). Building on our evolutionary analysis of MIK2 and SCOOPs, we hypothesized that the SCOOP SxS motif forms contact with the putative ligand-binding site predicted in our RCM analysis (Fig. 2*C*). AFM predicts a high interface predicted Template Modeling (ipTM > 0.84) score for 12 out of 50 *Arabidopsis* SCOOPs in complex with *Arabidopsis* MIK2 (Fig. 3*A*). Strikingly, the predicted interactions between SCOOP12 and MIK2 all fall within the strongly conserved putative functional sites for ligand recognition on the predicted surface of the LRRs, as delineated in our RCM analysis (Fig. 3*B*). Across species and SCOOPs, only the SxS motif is relatively strongly conserved. Moreover, single serine to alanine mutations in the SxS motif of SCOOP12 either reduce or abolish SCOOP12-induced ROS in *Arabidopsis* (14). In the AFM model, the SxS SCOOP motif is predicted to interact with the same two putative binding pockets on MIK2 across all twelve high-scoring AFM predictions. AF3 predictions confirmed this finding for 19 out of 50 predictions (45). The hereafter referred to as S5 and S7 MIK2 binding pockets engage in hydrogen bond interactions with S5 and S7 of the 13mer SCOOP peptide, and play a key role as recognition points for the peptide within the receptor. These pockets are composed by the MIK2-specific AAs D246/N268 and S292/H294/H316, respectively (Fig. 3*C*). Consistent with the AFM complex predictions, these two binding pockets are fully conserved within the earlier defined MIK2 clade as suggested by RCM. Additionally, AF predictions suggest that SCOOP peptides accommodate along the MIK2 inner binding canyon using a network of polar and nonpolar interactions that vary depending on the SCOOP sequence. We additionally attempted to predict a receptor/coreceptor/ligand complex for the 12 SCOOPs that resulted in a high ipTM with solely the receptor, again using just the ectodomains, but AFM failed to predict the tripartite MIK2–BAK1–SCOOP complex without any AA side chain clashes. In contrast, AF3 analysis of the tripartite complex resulted in 8 models that predict A) both the S5 and S7 binding pockets, B) consistent positioning of the full 13-mer SCOOP, and C) consistent positioning of BAK1 similar to the SERK1 orientation in the solved HSL1–IDL2–SERK1 complex (rmsd of 2 Å comparing 635 pairs of corresponding Cα atoms between the different complexes) (*SI Appendix*, Fig. S4). Moreover, these predictions suggest an interaction between the four C-terminal SCOOP AAs and the N-terminal loop of BAK1 (*SI Appendix*, Fig. S4*D*).

### Single and Double AA Changes within the AFM-Predicted Binding Pockets Affect Ligand-Induced ROS Production.

To test experimentally the biological relevance of the predicted binding pockets, we created constructs with single and double AA substitutions in both the S5 and the S7 MIK2 binding pockets. We expressed wild-type *Arabidopsis* MIK2 or binding pocket variants in *N. benthamiana,* and tested the capacity of the variants to perceive SCOOPs as measured by SCOOP-induced ROS production. Western blot analysis and confocal microscopy demonstrated that most variants accumulated to comparable protein levels with wild-type MIK2 and were correctly localized to the plasma membrane, respectively (*SI Appendix*, Fig. S5). One variant, H316G, exhibited altered subcellular localization and was therefore excluded from further experiments. The SCOOPs tested were selected from diverse *Arabidopsis* SCOOP clusters and are known to induce ROS production in *Arabidopsis* (17).

**Fig. 4.**
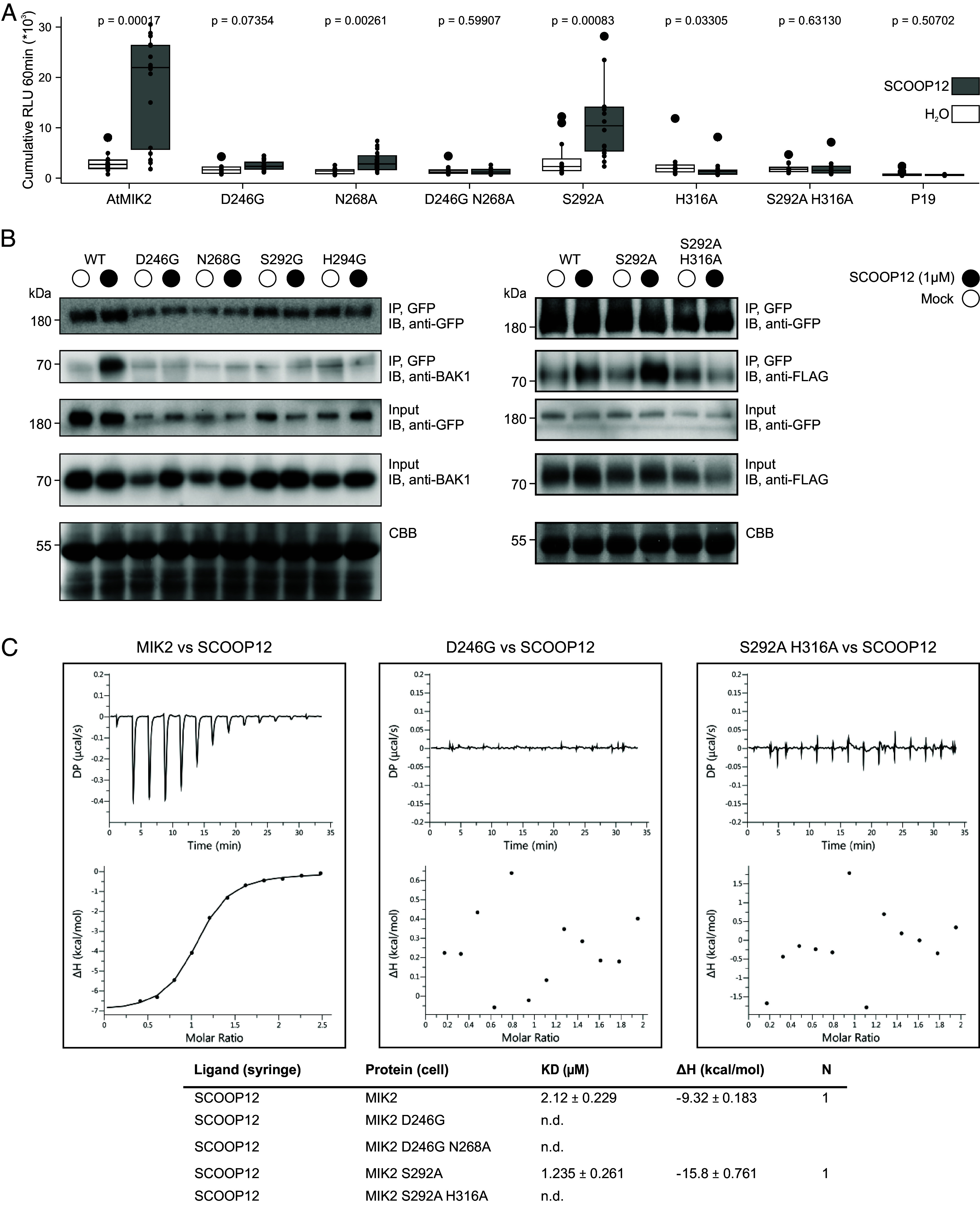
Single and double AA changes within the putative MIK2 binding pockets affect SCOOP12 ligand-binding, SCOOP12-induced MIK2–BAK1 complex formation, and ROS. (*A*) Shown is ROS production (4 to 60 min) in cumulative RLUs post treatment with H_2_O (white) or SCOOP12 (1 μM, gray). Four independent biological replicates (n = 4 plants) were performed, with each biological replicate represented by four technical replicates. Significance was tested by performing a paired Wilcoxon rank-sum test. (*B*) Coimmunoprecipitation post heterologous expression in *N. benthamiana* of BAK1 or BAK1-FLAG with MIK2-GFP after treatment with 1 μM SCOOP12, or water for 15 min. Western blots were probed with antibodies α-GFP and α-BAK1. (*C*) Isothermal titration calorimetry (ITC) experiments of MIK2 binding pockets variants vs. SCOOP12 and summary table. *K*_d_ (dissociation constant) indicates the binding affinity between the two molecules considered (in micromolar). The N indicates the reaction stoichiometry (*n* = 1 for a 1:1 interaction). The values indicated in the table are the mean ± SD of at least two independent experiments. N.d. = nondetected binding.

Intriguingly, single AA changes in the S5 binding pocket consistently reduced or abolished MIK2 function (Fig. 4*A* and *SI Appendix*, Fig. S6). The D246G MIK2 variant abolished ROS production in response to 7/8 tested SCOOPs, whereas N268G and N268A abolished ROS in response to 7/8 and 4/6 tested SCOOPs, respectively. The double mutant in the S5 binding pocket, D246G N268A, abolished ROS production for all 6 SCOOPs tested. In contrast, considering the S7 binding pocket, S292A from the S7 binding pocket still significantly induces ROS production in response to 5 out of 6 SCOOPs tested. H294G abolished ROS production in response to only 4 out of 8 tested SCOOPs. Nevertheless, H316A and the double S7 binding pocket mutant S292A H316A abolished ROS production for all 6 SCOOPs tested.

### Single AA Changes within the AFM-Predicted Binding Pockets Affect MIK2–BAK1 Complex Formation.

Like many other LRR-RKs, ligand-binding to MIK2 triggers complex formation with coreceptor kinases from the SOMATIC-EMBRYOGENESIS RECEPTOR-LIKE KINASE (SERK) family, primarily BAK1 (15, 18). A coimmunoprecipitation assay was performed after heterologous expression in *N. benthamiana* to test whether MIK2 variants could form a complex with BAK1 following SCOOP perception (Fig. 4*B*). Relative to the corresponding mock treatments, a clear induction of complex formation could be observed for both MIK2–BAK1 and S292A-BAK1 post SCOOP12 treatment. This was not the case for the other tested MIK2 variants. However, it is important to highlight that this is a semiquantitative assay, and we cannot exclude that complex formation for MIK2 variants happens at a lower level relative to WT MIK2.

### Single AA Changes within the AFM-Predicted Binding Pockets Affect Ligand-Binding.

To investigate the relevance of the S5 and S7 MIK2 binding pockets in the anchoring and recognition of SCOOP12 to the receptor; we produced recombinant ectodomains of MIK2 binding variants in both pockets, using insect cell cultures. These variants were then subjected to direct isothermal titration calorimetry (ITC) experiments with synthetic SCOOP12. We designed and tested the following mutants in the distinct MIK2 binding pockets: D246G and D246G/N268A in the S5 pocket and S292A and S292A/H316A in the S7 pocket. Due to the incorrect localization observed in the full-length H316G MIK2 mutant in the S7 binding pocket in *N. benthamiana* (*SI Appendix*, Fig. S5), we generated a variant where H316 was substituted with alanine (A). All expressed MIK2 mutants exhibited proper folding and eluted as monomers in size-exclusion chromatography experiments (*SI Appendix*, Fig. S7). To evaluate the interaction between MIK2 pocket variants and SCOOP12, we titrated the peptide into a solution containing the isolated MIK2 ectodomain variants. We did not detect any binding of SCOOP12 to the double MIK2 mutant D246G/N268A in the S5 binding pocket (Fig. 4*C* and *SI Appendix*, Fig. S8). We next assessed the individual contribution of the core S5 binding pocket residue D246. ITC experiments revealed that mutation of D246 alone is sufficient to disrupt the anchoring and recognition of SCOOP12 by MIK2 (Fig. 4*C* and *SI Appendix*, Fig. S8). In contrast, the single mutation S292A in the S7 pocket retained the ability to bind the peptide with WT affinity [Fig. 4*C*, (18)]. However, when the S292A mutation was combined with H316A, the interaction with SCOOP12 was completely lost (Fig. 4*C* and *SI Appendix*, Fig S6). These in vitro biochemical data therefore confirm our computational prediction as well as validated in vivo biochemical and physiological data.

## Discussion

Secreted signaling peptides regulate growth, development, and stress responses. In this study, we described the evolution of a lineage-specific peptide family and its receptor. Subsequently, we leveraged the acquired receptor/peptide homologues in combination with AI-driven protein structural prediction to unravel the mechanism of ligand-binding. Our approach paves the way for rapid identification of peptide–receptor interaction mechanisms.

Initially, *PROSCOOP12* was identified in *Arabidopsis* by analysis of transcriptomic profiles upon exposure to stresses (14). A subsequent screening of the genome revealed a novel peptide family that resides on just two loci that harbor 14 homologous genes with a similar intron–exon structure. Seven additional species were mined for putative homologues using a BLASTP approach, resulting in 74 putative *PROSCOOPs* within the Brassicaceae (14). The list of *Arabidopsis* *PROSCOOPs* was then extended to 23 and then 28 members (15, 16). However, a recent comprehensive bioinformatic analysis brought the total number to 50 diverse *PROSCOOPs* (17). It is generally difficult to identify peptide homologues from distantly related species by BLAST, due to the high sequence variability of prepropeptides, except the short region encoding the mature peptide. Hence, it is advised to limit the query to the most conserved part of the prepropeptide (2). Last, due to the low similarity between putative homologues, it is unclear whether they are true homologues, or just sequences that evolved independently (50). Therefore, this study leveraged a locus analysis (i.e., ~evolutionary linkage), which facilitated subsequent HMM searches with optimized queries across 350 predicted proteomes covering the plant kingdom. This effort resulted in 1,097 putative *PROSCOOPs* (Dataset S1), transcending the Brassicaceae family, but limited to the order of the Brassicales. Hence, the SCOOP family, like it is proposed for the systemin family, is evolutionarily young relative to two other well-characterized stress-related secreted signaling peptide families, PIPs and CTNIPs/SCREWs, which were identified across a multitude of diverse Angiosperms (2, 51–54). However, in contrast to systemin (54), the SCOOP family is ubiquitously present within the lineage they occur.

Most putative Brassicales SCOOPs have the previously described SxS motif (Fig. 1), and serine to alanine mutations in SCOOP12 highlighted the importance of these two serine residues for SCOOP perception (14). The double S5A/S7A and single S5A mutation did not induce ROS production whereas the S7A mutation resulted in a low, but still significant ROS production. Moreover, ITC analysis showed that the MIK2 ectodomain binds SCOOP12 but not the double S5A/S7A SCOOP12 variant (15). The SCOOP SxS motif is unique across known plant-secreted signaling peptides in contrast to the N-terminal asparagine and sulfated tyrosine motif found in RGF, PSY, and CIF peptides and the core PSGP sequence of the proline-rich CLE, CTNIP, PIP/PIPL, CEP, and IDA/IDL peptides (55). In contrast to the broader SCOOP family, certain individual SCOOPs show a strong conservation across the length of the predicted active peptide (Fig. 1*B* and *SI Appendix*, Fig. S2*B*), suggesting a conserved function across the species in which they were identified.

Although evolutionary analysis of secreted signaling peptide receptors has been reported previously (51, 56), earlier studies lacked the depth required to study the emergence of specific receptor functions and facilitate their mechanistic understanding (32, 57). Moreover, with more genomes sequenced, there is presently great opportunity to explore peptide signaling beyond model species (55). We measured SCOOP-induced ROS production across the order of the Brassicales and observed it in all species tested. In contrast, *N. benthamiana* and *Solanum lycopersicum* from the Solanaceae family are nonresponsive to SCOOP12 treatment (14).

We complemented phenotypic observations with a multitude of in silico approaches to unravel the evolutionary gain of MIK2 and SCOOP perception. Analysis of the *MIK2* locus across 32 species facilitated an HMM search across 350 predicted proteomes. Phylogenetic analysis using the kinase domains of the resulting LRR-RKs delineated a monophyletic clade, which contained all previously identified putative MIK2 homologues. After manual curation—as it is important to filter for an equal amount of LRRs when identifying LRR receptor homologues (2)—a maximum-likelihood phylogeny was performed, which ultimately revealed a putative MIK2 clade containing 37 LRR-RKs from 31 species including *A. thaliana* MIK2. The putative MIK2-paralogue clade is the closest related clade to the putative MIK2 clade, contains 15 putative MIK2 paralogues—all residing outside the contiguous MIK2 locus—and are only found in species that also contain a putative MIK2 homologue. Finally, we performed a RCM analysis on both clades of putative MIK2 homologues and paralogues (48). RCM predicts functional sites in LRR domains using signatures of conservation/diversification of surface residues, given a group of receptor homologues as an input. Hence, the presence of shared predicted functional sites within putative receptor homologues such as the putative MIK2 clade is an indicator that they might share a conserved function.

LRR-RKs of the putative MIK2 clade of four Brassicaceae genera were tested for SCOOP responsiveness and could induce ROS production and elevated cytosolic Ca^2+^ concentrations upon heterologous expression in *N. benthamiana*. Intriguingly, none of the investigated Brassicaceae lacks a MIK2 homologue within the MIK2 clade, suggestive of a strong conservation subsequent to the evolutionary gain of SCOOP perception. Neither MIK2 homologues, nor putative MIK2 paralogues, were identified outside the Brassicales. Hence, combining the results of the in silico analysis of SCOOPs and MIK2, the native plant responses of Brassicales, and the response of diverse MIK2 homologues post heterologous expression upon treatment with SCOOPs, we suggest the appearance of ancestral SCOOPs and an ancestral MIK2 at least ~39 Mya (47). However, more genome assemblies of species that diverged relatively closely to the divergence of the Brassicales species *C. violacea* are crucial to resolve the exact sequence of these events.

Additionally, our functional analysis of MIK2 homologues in a heterologous model (*N. benthamiana*) provides insight into LRR-RK function as it facilitated an in-depth RCM analysis. Additional sequences increase the reliability and power of RCM analysis. Hence, using 37 MIK2 homologues, this resulted in a clear distinction between conserved and diversified areas. Conserved regions on the surface of folded proteins often correspond to key functional sites such as for example ligand-binding sites (48, 58, 59). Nevertheless, not all interacting residues of LRR receptors and ligands are necessarily conserved across homologues as they might confer specificity. For example, although present across species, neither tested CTNIPs nor PEPs are recognized across species boundaries (a phenomenon referred to as conspecificity), putatively due to coevolution of the ligand and its receptor (51, 56). Additionally, not all high-scoring RCM residues interact with ligands based on the available structural data of receptor–ligand complexes (48). Last, interpretation of RCM analysis is easier with the help of a protein structural model. Therefore, we opted to combine this strategy with the use of AI-driven protein structural prediction with AFM and AF3. The conserved SCOOP SxS motif was predicted to interact with two conserved binding pockets within all MIK2 homologues. Moreover, MIK2 residues predicted to interact with the 13mer SCOOP12 all fall within conserved RCM residues (Fig. 3*B*). Hence, these two diverse approaches strengthen each other’s predictions as they point toward the same putative binding area.

The S5 and S7 binding pockets were functionally validated by testing MIK2 variants using three experimental approaches; A) SCOOP12-binding to MIK2 ectodomain in vitro, B) SCOOP12-induced complex formation with its coreceptor BAK1 in vivo, and C) ROS production upon SCOOP treatments as a marker for receptor complex activation (Fig. 4 and *SI Appendix*, Fig. S6). For example, relative to WT MIK2, a single AA mutation of D246 within the S5 binding pocket abolished direct SCOOP12-binding, diminished complex formation with BAK1 upon SCOOP12 exposure and abolished ROS production upon heterologous expression in response to eight diverse SCOOPs. In contrast, the S292A mutation within the S7 binding pocket did not abolish SCOOP12-binding in vitro but the double mutation S292A H316A did. Moreover, the two single mutations S292A and H294G in the S7 binding pocket have less drastic impact on ROS production to certain SCOOPs. This aligns with their relatively distant position to S7 of 3.4 Å and 3.2 Å, respectively. Overall, this suggests that complex formation with BAK1 might still happen depending on the interacting SCOOP and the specific mutation, as shown in the coimmunoprecipitation assay between S292A, BAK1, and SCOOP12. Importantly, these results are consistent with single replacements to alanine within the SxS motif of SCOOP12. Whereas SCOOP12 S5A does not induce ROS production in *Arabidopsis* Col-0, SCOOP12 S7A shows a comparatively low but significant ROS burst (14). Finally, although most putative SCOOPs contain the SxS motif, a minority harbor an “SxT” motif instead (Fig. 1). Therefore, we hypothesize that the S5 binding pocket functions as an anchor point by initiating SCOOP binding through S5 of the SxS motif. Subsequently, the S7 binding pocket most likely stabilizes SCOOP binding. Intriguingly, in contrast to other peptide families that can be perceived by several phylogenetically related LRR-RKs, perception of the 50 predicted SCOOPs seems to solely necessitate MIK2 (17, 18). AF3 predicts interaction between the four C-terminal SCOOP AAs, which are diverse across SCOOPs (Fig. 1), and the N-terminal loop of BAK1 in the MIK2–SCOOP–BAK1 complex (*SI Appendix*, Fig. S4*D*). Combined with the differentially affected ROS production by MIK2 variants following exposure to certain SCOOPs (*SI Appendix*, Fig. S6), this suggests that specific SCOOP-induced responses might partly rely on divergent binding and MIK2–SCOOP–BAK1 complex formation besides potential transcriptional and spatial regulation (60).

In this study, beyond deciphering SCOOP/MIK2 coevolution and SCOOP–MIK2 binding mechanisms, we pioneered the use of AI-driven protein complex prediction by AFM and AF3 in combination with comparative genomics to identify ligand-binding pockets for a peptide–receptor pair. The success of our approach depends on the accuracy of the complex prediction, which remains a challenge relative to monomer predictions (42). Not surprisingly, using the standard AFM approach, the interaction of the SxS motif of 12 out of 50 SCOOPs with MIK2 was predicted correctly. Nevertheless, results of the 5^th^ joint CASP-CAPRI protein assembly prediction challenge indicate a remarkable improvement of complex predictions relative to the 4^th^ meeting 2 y prior (42). Moreover, a multitude of participating groups exceeded the performance of the benchmark standard, AFM. This trend was also observed for the AF3 predictions included in this study: seven additional SCOOPs, 19/50 in total, resulted in correct predictions of the MIK2–SCOOP binding pockets relative to AFM (Fig. 3*B*). Moreover, based on comparative structure analysis with resolved tripartite receptor complexes such as HSL1–IDL2–SERK1 (*SI Appendix*, Fig. S4*E*), a multitude of promising predictions of the MIK2–SCOOP–BAK1 complex were obtained using AF3, whereas AFM failed consistently (*SI Appendix*, Fig. S4). Hence, AI-driven complex predictions are improving and thus will play an important role in unraveling other peptide–receptor interactions in the future.

## Material and Methods

Detailed material and methods for this study can be found in *SI Appendix, Supporting Text; Material and Methods*. First, computational analyses are described: this includes mining and analysis of PROSCOOPs and MIK2, maximum-likelihood phylogeny, RCM, Brassicales species tree, AFM, and AF3 and structural visualization and model analysis. Second, *SI Appendix* contains the plant materials and synthetic peptides used. Third, detailed description of the following experimental procedures: SCOOP-induced ROS production, molecular cloning, transient expression—ROS measurements—cytoplastic calcium measurements in *Nicotiana benthamiana*, protein extraction and western blotting, protein expression and purification, isothermal titration calorimetry (ITC), analytical size-exclusion (SEC) chromatography, and coimmunoprecipitation (Co-IP). Primers used in this study are available in *SI Appendix*, Table S1. Datasets S1–S3.

## Supplementary Material

Appendix 01 (PDF)

Dataset S01 (XLSX)

Dataset S02 (XLSX)

Dataset S03 (TXT)

## Data Availability

Plasmid maps, predicted protein structures, and source files of blots (Datasets S4–S6) have been deposited in Zenodo (DOI: 10.5281/zenodo.11615633) (61). All other data are included in the article and/or supporting information.
