# Supplementary material for: Leveraging coevolutionary insights and AI-based structural modeling to unravel receptor–peptide ligand-binding mechanisms
Source: Proc Natl Acad Sci U S A. 2024 Aug 6;121(33):e2400862121. doi: 10.1073/pnas.2400862121 (PMC11331138; doi:10.1073/pnas.2400862121)
Supplement: Supplementary file 1 — Appendix 01 (PDF) [file pnas.2400862121.sapp.pdf]

PNAS

## Supporting Information for

# Leveraging co-evolutionary insights and AI-based structural modeling to unravel receptor-peptide ligand-binding mechanisms

Simon Snoeck, Hyun Kyung Lee, Marc W. Schmid, Kyle W. Bender, Matthias J. Neeracher, Alvaro D. Fernández-Fernández, Julia Santiago, Cyril Zipfel

Corresponding authors: Simon Snoeck and Cyril Zipfel
Email: simon.snoeck@uzh.ch and cyril.zipfel@uzh.ch

**This PDF file includes:**

Supporting text; material and methods
Figures S1 to S8
Table S1
Legends for Datasets S1 to S6

**Other supporting materials for this manuscript include the following:**

Datasets S1 to S3 (separate files)
Datasets S4 to S6 (10.5281/zenodo.11615634)

# Supporting text; Material and Methods

## *PROSCOOPs*

### Mining and analysis

First, a locus analysis was performed for each Arabidopsis *PROSCOOP* locus across the Brassicales as described before (1, 2). The analyzed genome assemblies, versions, and their sources can be found in Dataset S2. In short, BLASTP (BLAST 2.9.0+, e-value 10) was used to identify *PROSCOOP* syntenic loci by mining the genomes for homologues of the strongly conserved neighbor genes of Arabidopsis *PROSCOOPs* which can be found in Dataset S2. Second, the novel candidate PROSCOOPs were added to the earlier identified 50 Arabidopsis PROSCOOPs and clustered with MMseqs2 (release 14-7e284) using a minimal sequence identity and coverage of 0.5 and 0.3, respectively (3). Third, for each cluster with at least three sequences, we built an HMM profile by aligning the sequences with muscle and running hmmbuild (4, 5). These profiles were used to search for additional *PROSCOOP* candidates in a collection of genomes from 350 species described previously (6). The results were filtered for an e-value below 10e-5, resulting in a total of 1168 *PROSCOOP* candidates, all are limited to Brassicales. Finally, putative SCOOPs were extracted based on the corresponding 13- and 15mer of the Arabidopsis SCOOPs if present in the cluster, otherwise the position of the 'SxS' motif was used to predict a 13mer. Putative active SCOOPs were extracted for 1097 out of 1168 sequences. Seventy-one candidate *PROSCOOP*s were filtered out due to irregularities in the alignment potentially caused by ORF shifts due to sequencing errors, incorrect annotations, or pseudogenes. Gene accessions, protein sequences and predicted 13-mers of all putative *PROSCOOPs* can be found in Dataset S1.

## MIK2

### Mining and analysis

Similar to the *SCOOP*s, the analyzed genome assemblies, versions, and their sources included in the contiguous *MIK2* locus analysis can be found in Dataset S2. Putative MIK2 homologues were identified using locus analysis within 32 Brassicales species. In short, BLASTP (BLAST 2.9.0+, e-value 10) was used to identify the *MIK2* syntenic locus and putative MIK2s by mining the genomes initially for homologues of the strongly conserved neighbor (anchor) genes of Arabidopsis *MIK2* (*AT4G08850*); *AT4G08810*/*AT4G08840* and *AT4G08870*/*AT4G08920*. Subsequently, putative MIK2 homologues were identified using BLASTP, resulting in a list of 39 putative MIK2 homologues. Locus comparison was performed using R (v4.0.3) and the R-package genoPlotR (v0.8.11) using the extracted contiguous *MIK2* loci and their corresponding annotation (Dataset S2). The resulting figure was edited in Corel-DRAW Home & Student x7.

Subsequently, we built an HMM profile with hmmbuild (version 3.1b2) and searched all proteins from the collection of 350 genomes that were longer than 300 AA for matches (hmmsearch --max -E1e-10) (5). Given the relaxed e-Value threshold, we found 435,387 initial matches which is close to what one would find with a plain protein-kinase HMM from PFAM. We then filtered for matches with an e-value below 1e-250, thus reducing the set to 4,786 candidates. All initial MIK2 homologues identified through the synteny approach were included in the novel list. We then extracted the kinase domain using hmmsearch and the kinase PFAM pattern PF00069.26. For each sequence, we selected the best matching stretch and extracted the sequence with bedtools (version 2.92.2) (7). Sequences were then aligned with FAMSA (version 1.6.2) (8). Alignments were not trimmed, and phylogenetic trees were inferred with FastTree (version 2.1.11 SSE3, option -lg) (9, 10). Finally, we extracted the clade that contained all initial candidates with gotree (version 0.4.0, github.com/evolbioinfo/gotree).

### Maximum-likelihood Phylogeny

The above set of putative *MIK2* homologues and potential *MIK2* paralogues was filtered for potential pseudogenes, distinct LRR-RKs and wrongly annotated genes using the following filters: A) candidate sequence with a sequence

length > 90 % and < 110 % of Arabidopsis *MIK2* were retained, B) sequences with gaps or inserts within strongly conserved domains were removed. Initial alignments were performed using the online version of MAFFT 7 using the E-INS-i strategy (Dataset S3), the L-INS-I strategy was preferred once major gaps were removed in the alignment (11). A phylogenetic analysis was performed on the CIPRES web portal using RAXML-HPC2 on XSEDE (v8.2.12) with the automatic protein model assignment algorithm using ML criterion and 250 bootstrap replicates (12, 13). The JTT likelihood protein model was selected as the best scoring model for ML analysis. The resulting tree was rooted using an LRR-RK found at the conserved MIK2 locus in *T. cacao*, visualized using MEGA10, and edited in Corel-DRAW Home & Student x7. The above strategy was repeated with just the extracellular domains (14).

## RCM

RCM plots were created as previously described (15). In short, we extracted the LRR domains with predict-phytolrr (obtained in December 2021) (16), aligned them with muscle (version 3.8.31, (4)), trimmed gaps with trimal (version v1.4.rev22) (17), calculated conservation scores with mstatx (1-weighted entropy, github.com/gcollet/MstatX), and extracted a consensus sequence with em_cons (18). To plot the conservation score onto the potential three-dimensional structure, positions of the LRR repeats were again identified with predict-phytolrr.

## Brassicales species tree

The tree shown in Fig. S1 was extracted from a larger tree that spanned the 350 plant species previously described(6, 19) . To construct the original phylogenetic tree with 350 species, we adapted a previously described protocol (20). First, we searched single-copy genes with BUSCO using the viridiplantae_odb10 database (version 5.5.0) (21). No gene from the BUSCO database was found in all species (the maximum was 300 species at once). We thus used 425 BUSCO-genes that occurred in more than 200 species. For each gene, we extracted all sequences, aligned them with muscle (4), trimmed the alignment with trimal (version 1.4.rev22, option -gt 0.9) (17), and constructed a tree with FastTree (version 2.1.11 SSE3, option -lg) (9). Trees from all genes were finally merged into a single tree that included all species using ASTRAL (version 5.6.3) (22). The final tree was rooted with *C. paradoxa*. Subsequently, the tree was converted to an ultrametric tree using the function chronos() from the R-package "ape" (version 5.6-2) (22). Ultimately, for Fig. S1, we extracted the branch that includes the Brassicales as *PROSCOOPs* are limited to this order and retained *C. papaya* as the closest outgroup. The resulting phylogeny was verified by comparison with the work of Hendriks et al. (2023) (23).

## Plant materials and synthetic peptides

*Arabidopsis thaliana* ecotype Columbia (Col-0) was used as wild-type control for SCOOP-induced ROS production and were, similar to other *Brassicales*, grown in growth chambers (20 °C, 60 % RH and 10:14 light:dark cycles). Other plants tested were: *Carica papaya* Tainung, *Cleome violacea* N29053, *Eutrema salsugineum* N22504, *Euclidium syriacum* GC 0587-68, *Diptychocarpus strictus* KM 05-0397-10-00, *Brassica rapa* R500. *Nicotiana benthamiana* was used for experiments which leveraged heterologous expression and were grown in the greenhouse (25/22 °C day/night, 60 % RH and 16:8 light:dark cycles). All synthetic peptides were ordered at >80 % purity (physiological assays) or >95 % purity (biochemical assays) (EZBiolabs) and were earlier described (1).

## SCOOP-induced ROS production within and outside the Brassicales

All peptides used in this study were synthesized and reconstituted in $H_2O$. SCOOP12 (PVRSSQSSQAGGR), SCOOP16 (YVPPSKSRRGKGP), SCOOP21 (YVPPSKSRRGKGP) and SCOOP24 (RVPRSKSPPDRQW) were leveraged to test the activity of putative MIK2 homologues. The flg22 peptide (sequence QRLSTGSRINSAKDDAAGLQIA) originates from bacterial flagellin and was used as a positive control for ROS production upon peptide treatment (24). Leaf punches were taken with a 4-mm biopsy punch and floated in 100 µL of $H_2O$ using individual cells of a white 96-well white bottom plate (Greiner F-Boden, lumitrac, med. Binding, [REF 655075]). After overnight incubation, $H_2O$ was

removed and ROS production was measured upon addition of a 100-µL assay solution which contained 10 µg/mL horseradish peroxidase (P6782, Merck), 10 mM luminol and the treatment (1 µM SCOOP, 0.1 µM flg22 or $H_2O$). Luminescence was quantified with a HIGH-RESOLUTION PHOTON COUNTING SYSTEM (HRPCS218, Photek). Four technical replicates per biological replicate (specified in Fig. S2) were quantified for each treatment, significant differences were determined by performing a two-group Mann-Whitney U Test between each SCOOP treatment and the mock treatment. R and the R-packages dplyr (v1.1.2), ggpubr (v.0.6.0), and ggplot2 (v3.4.2) were used to analyze and plot the data. The resulting figure was edited in Corel-DRAW Home & Student x7.

## Functional validation MIK2 homologues

### Molecular cloning

All constructs were created using a hierarchical modular cloning approach facilitated by the MoClo toolkit and the MoClo Plant Parts kit (25, 26). For recombinant expression, we used the previously published Arabidopsis *MIK2* sequence (L0 level backbone, CZLp4829) (27), and *MIK2* homologues were synthesized with domesticated BsaI and BpiI sites and inserted in a pMA-RQ backbone (Invitrogen, Thermo Fisher Scientific). Subsequently, the L0 fragments and an mEGFP C-terminal tag (CZLp4772) were inserted into level 1 Golden-Gate plasmids CZLp4130, which already includes a 35S promotor (CaMV) and an OCS terminator. GoldenGate reactions were performed with 5 U of restriction enzyme, 200 U of T4 ligase in T4 ligase buffer (NEB), 0.1 mg/mL BSA (NEB) and 40 GoldenGate digestion ligation cycles (25). All constructs were validated by Sanger sequencing upon completion and plasmid maps can be found in Dataset S4 (Eurofins genomics).

### Transient expression in *Nicotiana benthamiana*

*N. benthamiana* does not respond to SCOOP12, allowing the use of heterologous expression in *N. benthamiana* to test putative MIK2 function (28). *Agrobacterium tumefaciens* strain GV3101 transformed with the appropriate construct were grown overnight in LB-media and spun-down. The bacteria were resuspended in infiltration media (10 mM MES-KOH, pH 5.8, 10 mM $MgCl_2$) and adjusted to an $OD_{600}$ of 0.5. After 3 h of incubation, the youngest fully expanded leaves of 4- to 5-week-old plants were infiltrated.

### ROS measurements in *Nicotiana benthamiana*

Following Agrobacterium infiltration for receptor expression (24-48 h), leaf punches were taken with a 4-mm biopsy punch and floated in 100 µL of $H_2O$ using individual cells of a white 96-well white bottom plate (Greiner F-Boden, lumitrac, med. Binding, [REF 655075]). Subsequently, the same procedure was followed as outlined before while using diverse Arabidopsis SCOOPs (AA sequences, Dataset S1) and flg22. Biological replicates were quantified (n≥4 plants), with each biological replicate representing four technical replicates. R and the R-packages dplyr (v1.1.2), ggpubr (v.0.6.0), and ggplot2 (v3.4.2) were used to analyze and plot the data. The resulting figure was edited in Corel-DRAW Home & Student x7.

### Cytoplastic calcium measurements in *Nicotiana benthamiana*

Following Agrobacterium infiltration for receptor expression (24 h) in a stable aequorin expressing line of *N. benthamiana* (29), leaf punches were taken with a 4-mm biopsy punch and floated in 100 µL of $H_2O$ with 20 µM coelenterazine (Merck), using individual cells of a white 96-well white bottom plate (Greiner F-Boden, lumitrac, med. Binding, [REF 655075]). After overnight incubation, the coelenterazine solution was replaced with 100 µL $H_2O$ and rested for a minimum of 30 min in the dark. Two readings were taken in a TECAN SPARK plate reader every minute for 45 min using an integration time of 250 ms. Biological replicates were quantified (n=4 plants), with each biological replicate representing four technical replicates. R and the R-packages dplyr (v1.1.2), ggpubr (v.0.6.0), and ggplot2 (v3.4.2) were used to analyze and plot the data. The resulting figure was edited in Corel-DRAW Home & Student x7.

## Protein extraction and western blotting

*N. benthamiana* leaf tissues were flash-frozen in liquid nitrogen and grounded using plastic pestles in 1.5-mL microcentrifuge tubes. Grounded tissue was mixed with 2× loading sample buffer (4 % SDS, 20 % glycerol, 20 mM DTT, 0.004 % bromophenol blue, and 100 mM Tris-HCl pH 7.5) for 10 min at 95 °C. Subsequently, samples were spun at 13,000 × g for 2 min prior to loading and running on a 1.5-mm 10 % SDS-PAGE gels. Proteins were transferred onto PVDF membrane (ThermoFisher) prior to incubation with α-GFP (B-2) HRP (Santa Cruz 9996 HRP, 1:1500). Western blots were imaged with a Bio-Rad ChemiDoc and Image Lab Touch Software (v2.2.0.08). Protein loading was visualized by staining the blotted membrane with Coomassie brilliant blue.

## AlphaFold-Multimer (AFM) and AlphaFold 3 (AF3)

AFM and AF3 protein structure complex predictions of the extracellular domain MIK2 (AT4G08850), all 50 Arabidopsis SCOOPs, with or without the extracellular domain of BAK1 (AT4G33430) were respectively created using the ColabFold platform (v1.3.0) and AlphaFold Server Beta (1, 30, 31). The extracellular domain of MIK2 and BAK1 were determined using deepTMHMM (14). The AF-multimer input sequence alignment was generated through MMseqs2 using the unpaired+paired mode without using templates (30, 32–34). Three recycles were run for each of the five created models. The five resulting models were ranked based by AFM (0.8*ipTM + 0.2*predicted Template Modelling (pTM) score). The structure files (.pdb) of the successfully predicted complexes and the corresponding predicted aligned error (PAE) files for AFM predictions are provided in Dataset S5. R and the R-packages dplyr (v1.1.2), ggpubr (v.0.6.0), and ggplot2 (v3.4.2) were used to analyze and plot the ipTM data. The resulting figures were edited in Corel-DRAW Home & Student x7.

## Structural visualization and model analysis.

MIK2-SCOOP prediction models obtained from AFM and AF3 were superimposed using UCSF Chimera (35). Molecular diagrams have been prepared with PyMOL (36), retrieved from http://www.pymol.org/pymol). Comparative structural analysis of MIK2-SCOOP-BAK1 and other receptor complexes was performed with COOT(37).

## Mutagenesis

All primers and plasmids used and generated in this study are listed (Table S1, Dataset S4). Site-directed mutagenesis (SDM) was conducted as described by (38). The L0 construct of Arabidopsis *MIK2* was used as template (CZLp4057 (27)). The PCR reaction was DpnI (New England Biolabs) digested at 37 °C for 2 h without prior clean-ups, and then transformed into *E. coli* DH10b. Similar as described before, the L1 constructs were completed by insertion into a level 1 Golden-Gate plasmid CZL4130, which already includes a 35S promotor (CaMV) and a NOS terminator, and the addition of an mEGFP C-terminal tag (CZLp4772). GoldenGate reactions were performed with 5 U of restriction enzyme, 200 U of T4 ligase in T4 ligase buffer (NEB), 0.1 mg/mL BSA (NEB) and 40 GoldenGate digestion ligation cycles (25). All constructs were validated by whole plasmid sequencing, plasmid maps can be found in Dataset S4 (Eurofins genomics).

## Confocal imaging

Protein localization in *N. benthamiana* was analyzed three days after transient infiltration with *A. tumefaciens* strains expressing different MIK2 variants tagged with C-terminal GFP. Fluorescence detection was performed in a Leica Stellaris with wavelength emission at 488 nm and detection at 494-560 nm for GFP and 625-750 nm range for chlorophyll autofluorescence. Images were modified using FIJI using the same parameters. For plasmolysis, leaf disks were pretreated with 0,6 M mannitol for 1 hour. Experiments were repeated 3 times with reproducible results.

## Protein expression and purification

*Spodoptera frugiperda* codon-optimized synthetic genes (Invitrogen GeneArt), coding for *Arabidopsis thaliana* MIK2 ectodomain (residues 1 to 709) mutants were cloned into a modified pFastBAC vector (Geneva Biotech) with its native signal peptide, a C-terminal TEV (tobacco etch virus protease) cleavable site and a StrepII-9xHis affinity tag. Baculovirus generation was carried out using DH10 cells and virus production and amplification was done in Sf9 cells. *Trichoplusia ni* Tnao38 cells were used for protein expression (39), that were infected with MIK2 mutant viruses with a multiplicity of infection (MOI) of 3 and incubated 1 day at 28°C and 2 days at 22°C at 110 rpm. The secreted proteins and complexes were purified by $Ni^{2+}$ (HisTrap excel, Cytiva, equilibrated in 25 mM $KP_i$ pH 7.8 and 500 mM NaCl) followed by Strep (Strep-Tactin Superflow high-capacity, IBA Lifesciences, equilibrated in 25 mM Tris pH 8.0, 250 mM NaCl, 1 mM EDTA) affinity chromatography. All proteins were incubated with TEV protease to remove the tags. Proteins were further purified by SEC on a Superdex 200 Increase 10/300 GL column (Cytiva) equilibrated in 20 mM citric acid pH5.0, 150 mM NaCl. Proteins were concentrated using Amicon Ultra concentrators (Millipore, molecular weight cut-off 3,000, 10,000 and 30,000), and SDS-PAGE was used to assess the purity and structural integrity of the different proteins.

## Isothermal titration calorimetry (ITC)

Experiments were performed at 25 °C using a MicroCal PEAQITC (Malvern Instruments) with a 200 µL standard cell and a 40 µL titration syringe. The MIK2 mutant ectodomains were gel filtrated into pH 5 ITC buffer (20 mM citric acid pH 5.0, 150 mM NaCl). SCOOP12 peptide powder was dissolved in the same buffer to obtain the desired concentration. A typical experiment consisted of injecting 3 µL of a 150 or 300 µM solution of the ligand into 15 µM MIK2 solution in the cell at 150 s intervals and 500 rpm stirring speed. ITC data were corrected for the heat of dilution by subtracting the mixing enthalpies for titrant solution injections into protein-free ITC buffer. Experiments were done in duplicates and data were analyzed using the MicroCal PEAQ-ITC Analysis Software provided by the manufacturer. All ITC runs used for data analysis had an N ranging between 0.8 and 1.3. The N values were fitted to 1 in the analysis.

## Analytical size-exclusion (SEC) chromatography

Analytical SEC experiments were performed using a Superdex 200 Increase 10/300 GL column (GE). The columns were pre-equilibrated in 20 mM citric acid pH 5, 150 mM NaCl. One hundred fifty micrograms of MIK2 mutant ectodomains were injected sequentially onto the column and eluted at 0.5 mL/min. Ultraviolet absorbance (UV) at 280 nm was used to monitor the elution of the proteins. The peak fractions were analyzed by SDS-PAGE followed by Coomassie blue staining.

## Co-immunoprecipitation (Co-IP)

Following Agrobacterium infiltration of receptor (variants), BAK1 (CZLp3593) and P19 (CZLp5085) expression (48 h), leaves were split in half and midveins were removed. This way, mock- and treatment of interest can later be performed on two samples created from the same infiltration event. All samples were submerged in 0.25x MS-sucrose for 30 min. Next, samples were vacuum infiltrated with 1 µM SCOOP12 in 0.25x MS-sucrose or just 0.25x MS-sucrose as a mock treatment. Finally, all samples were dried with paper towel and flash frozen.

For co-immunoprecipitation assays, approximately 3.5 g of frozen tissue was ground to a fine powder in nitrogen-cooled stainless-steel jars using a Retsch MM300 ball mill. Tissue was thawed in extraction buffer (50 mM Tris-HCl pH 7.5, 150 mM NaCl, 2 mM EDTA, 10 % (v/v) glycerol, 2 mM DTT and 1:100 home-made protease inhibitor cocktail equivalent to Sigma-Aldrich P9599) at a ratio of 2 mL of buffer per gram of tissue and proteins were solubilized on a rotator at 4 °C for 30 min. Extracts were filtered through two layers of Miracloth and centrifuged at 25,000 x g for 30 min at 4 °C to generate a clarified extract. Protein amounts were estimated using the Bradford assay and samples were normalized to contain equal amounts of protein.

Protein extracts containing GFP-tagged MIK2 or site-directed mutants were incubated with 20 µL of GFP-Trap beads (Chromotek) for 2 h with gentle mixing at 4 °C to immuno-precipitate receptor complexes. The beads were sedimented by centrifugation at 1000 x g for 4 min at 4 °C and were subsequently suspended in 1 mL of extraction buffer (see above). The beads were sedimented at 1,000 x g for 1 min and suspended in 1 mL of extraction buffer three more times for a total of four washes. After the last wash was removed, beads were suspended in 2X Laemmli SDS-PAGE loading buffer followed by heating at 80 °C for 10 min. Five microliters of each IP fraction was loaded into an 8 % (v/v) SDS-PAGE gel and proteins were separated for 90 min at 150 V followed by transfer to PVDF membrane for immunoblotting with anti-GFP (B-2) and anti-BAK1 antibodies.

# References


1. H. Yang, *et al.*, Subtilase-mediated biogenesis of the expanded family of SERINE RICH ENDOGENOUS PEPTIDES. *Nat Plants* **9**, 2085–2094 (2023).
2. S. Snoeck, N. Guayazán-Palacios, A. D. Steinbrenner, Molecular tug-of-war: plant immune recognition of herbivory. *Plant Cell* **34**, 1497–1513 (2022).
3. M. Steinegger, J. Söding, MMseqs2 enables sensitive protein sequence searching for the analysis of massive data sets. *Nat Biotechnol* **35**, 1026–1028 (2017).
4. R. C. Edgar, MUSCLE: multiple sequence alignment with high accuracy and high throughput. *Nucleic Acids Res* **32**, 1792–1797 (2004).
5. S. R. Eddy, Accelerated profile HMM searches. *PLoS Comput Biol* **7**, 1002195 (2011).
6. B. P. M. Ngou, R. Heal, M. Wyler, M. W. Schmid, J. D. G. Jones, Concerted expansion and contraction of immune receptor gene repertoires in plant genomes. *Nat Plants* **8**, 1146–1152 (2022).
7. A. R. Quinlan, I. M. Hall, BEDTools: a flexible suite of utilities for comparing genomic features. *BIOINFORMATICS APPLICATIONS NOTE* **26**, 841–842 (2010).
8. S. Deorowicz, A. Debudaj-Grabysz, A. Gudyś, FAMSA: Fast and accurate multiple sequence alignment of huge protein families. *Sci Rep* **6**, 1–13 (2016).
9. M. N. Price, P. S. Dehal, A. P. Arkin, FastTree 2 – Approximately maximum-likelihood trees for large alignments. *PLoS One* **5**, e9490 (2010).
10. G. E. Tan, *et al.*, Current methods for automated filtering of multiple sequence alignments frequently worsen single-gene phylogenetic inference. *Syst. Biol* **64**, 778–791 (2015).
11. K. Katoh, K. Misawa, K. Kuma, T. Miyata, MAFFT: a novel method for rapid multiple sequence alignment based on fast Fourier transform. *Nucleic Acids Res* **30**, 3059–3066 (2002).
12. M. A. Miller, W. Pfeiffer, T. Schwartz, Creating the CIPRES science gateway for inference of large phylogenetic trees in *2010 Gateway Computing Environments Workshop, GCE 2010*, (IEEE, 2010), pp. 1–8.
13. A. Stamatakis, RAxML version 8: a tool for phylogenetic analysis and post-analysis of large phylogenies. *Bioinformatics* **30**, 1312–3 (2014).
14. J. Hallgren, *et al.*, DeepTMHMM predicts alpha and beta transmembrane proteins using deep neural networks. *BioRxiv* 1–12 (2022). https://doi.org/10.1101/2022.04.08.487609.
15. L. Helft, *et al.*, LRR Conservation mapping to predict functional sites within protein leucine-rich repeat domains. *PLoS One* **6**, e21614 (2011).
16. T. Chen, Identification and characterization of the LRR repeats in plant LRR-RLKs. *BMC Mol Cell Biol* **22**, 1–16 (2021).
17. S. Capella-Gutiérrez, J. M. Silla-Martínez, T. Gabaldón, trimAl: A tool for automated alignment trimming in large-scale phylogenetic analyses. *Bioinformatics* **25**, 1972–1973 (2009).
18. P. Rice, L. Longden, A. Bleasby, EMBOSS: the European molecular biology open software suite. *Trends Genet* **16**, 276–277 (2000).
19. B. Pok, *et al.*, Evolutionary trajectory of pattern recognition receptors in plants. *bioRxiv* 2023.07.04.547604 (2023). https://doi.org/10.1101/2023.07.04.547604.
20. M. Manni, M. R. Berkeley, M. Seppey, E. M. Zdobnov, BUSCO: assessing genomic data quality and beyond. *Curr Protoc* **1** (2021).

21. M. Manni, M. R. Berkeley, M. Seppey, F. A. Simão, E. M. Zdobnov, BUSCO update: novel and streamlined workflows along with broader and deeper phylogenetic coverage for scoring of eukaryotic, prokaryotic, and viral genomes. *Mol Biol Evol* **38**, 4647–4654 (2021).
22. C. Zhang, M. Rabiee, E. Sayyari, S. Mirarab, ASTRAL-III: Polynomial time species tree reconstruction from partially resolved gene trees. *BMC Bioinformatics* **19**, 15–30 (2018).
23. K. P. Hendriks, *et al.*, Global Brassicaceae phylogeny based on filtering of 1,000-gene dataset. *Current Biology* **33**, 4052–4068 (2023).
24. G. Felix, J. D. Duran, S. Volko, T. Boller, Plants have a sensitive perception system for the most conserved domain of bacterial flagellin. *Plant J* **18**, 265–276 (1999).
25. E. Weber, C. Engler, R. Gruetzner, S. Werner, S. Marillonnet, A modular cloning system for standardized assembly of multigene constructs. *PLoS One* **6**, e16765 (2011).
26. C. Engler, *et al.*, A Golden Gate modular cloning toolbox for plants. *ACS Synth Biol* **3**, 839–843 (2014).
27. J. Rhodes, *et al.*, Perception of a divergent family of phytocytokines by the Arabidopsis receptor kinase MIK2. *Nat Commun* **12**, 1–10 (2021).
28. K. Gully, *et al.*, The SCOOP12 peptide regulates defense response and root elongation in *Arabidopsis thaliana*. *J Exp Bot* **70**, 1349–1365 (2019).
29. C. Segonzac, *et al.*, Hierarchy and roles of pathogen-associated molecular pattern-induced responses in *Nicotiana benthamiana*. *Plant Physiology Ò* **156**, 687–699 (2011).
30. M. Mirdita, M. Steinegger, J. Söding, MMseqs2 desktop and local web server app for fast, interactive sequence searches. *Bioinformatics* **35**, 2856–2858 (2019).
31. J. Abramson, *et al.*, Accurate structure prediction of biomolecular interactions with AlphaFold 3. *Nature* (2024). https://doi.org/10.1038/s41586-024-07487-w.
32. J. Jumper, *et al.*, Highly accurate protein structure prediction with AlphaFold. *Nature* **596**, 583 (2021).
33. M. Mirdita, *et al.*, Uniclust databases of clustered and deeply annotated protein sequences and alignments. *Nucleic Acids Res* **45** (2017).
34. A. L. Mitchell, *et al.*, MGnify: the microbiome analysis resource in 2020. *Nucleic Acids Res* **48**, D570–D578 (2020).
35. E. F. Pettersen, *et al.*, UCSF Chimera—A visualization system for exploratory research and analysis. *J Comput Chem* **25**, 1605–1612 (2004).
36. PyMOL, The PyMOL molecular graphics system, version 2.5.2 Schrödinger, LLC.
37. P. Emsley, B. Lohkamp, W. G. Scott, K. Cowtan, Features and development of Coot. *urn:issn:0907-4449* **66**, 486–501 (2010).
38. H. Liu, J. H. Naismith, An efficient one-step site-directed deletion, insertion, single and multiple-site plasmid mutagenesis protocol. *BMC Biotechnol* **8**, 1–10 (2008).
39. Y. Hashimoto, S. Zhang, S. Zhang, Y.-R. Chen, G. W. Blissard, Correction: BTI-Tnao38, a new cell line derived from *Trichoplusia ni*, is permissive for AcMNPV infection and produces high levels of recombinant proteins. *BMC Biotechnol* **12**, 12 (2012).

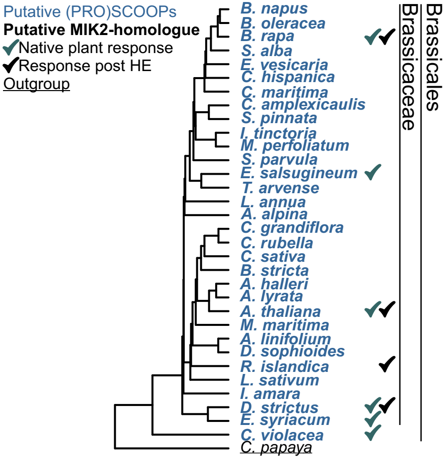


Fig. S1: **A species phylogeny that indicates besides the presence of putative SCOOP- and MIK2-homologues also SCOOP-induced plant-signaling responses in native plants and post heterologous expression (HE) in *N. benthamiana.***

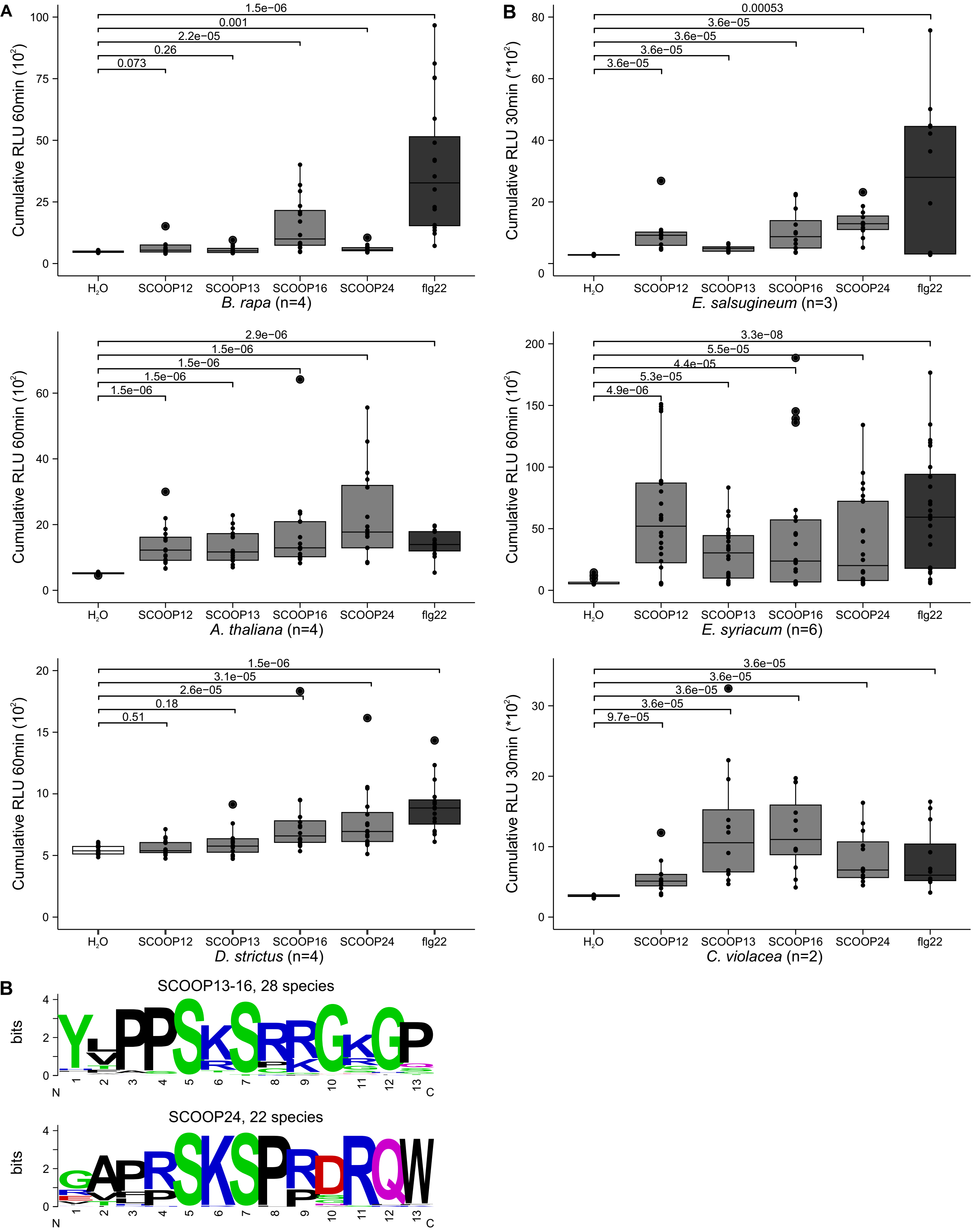


Fig. S2: **Diverse species of the order of the Brassicales respond to SCOOP treatment with reactive oxygen species (ROS) production. A)** Shown is ROS production in cumulative relative luminescence units (RLU) for (4-30 min or 4-60 min), four technical replicates per biological replicate (# indicated in the figure), in relative luminescence units (RLUs) (1 observation/min) after treatment with $H_2O$ (white), SCOOP12, SCOOP13, SCOOP16 and SCOOP24 (1 µM, grey) or the peptide flg22 (1 µM, dark grey). Significant differences between the control and the treatments of interest were found by performing a Wilcoxon rank-sum test. **B)** Sequence motif analysis of SCOOP13-16 and SCOOP24. Sequence logos were generated using Dataset S1 and WebLogo server (https://weblogo.berkeley.edu/logo.cgi).

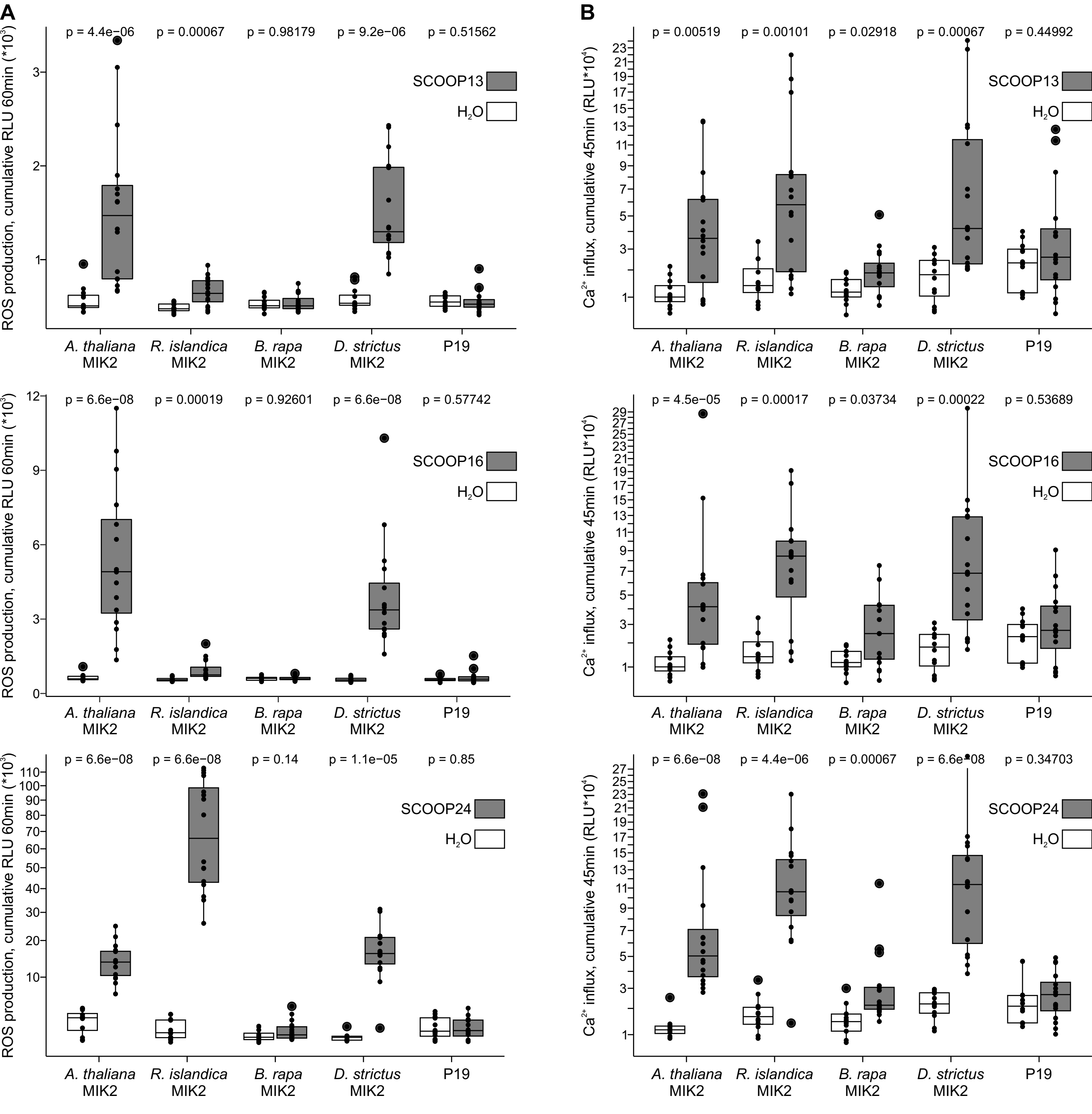


Fig. S3: **SCOOP-dependent reactive oxygen species (ROS) production and $Ca^{2+}$ influx following the heterologous expression of MIK2 and MIK2 homologues in *N. benthamiana*. A-B)** Shown are ROS production (A) and $Ca^{2+}$ influx (B), respectively 4-60 min and 3-45 min, in cumulative relative luminescence units (RLUs) post treatment with $H_2O$ (white) or SCOOP12, SCOOP13, SCOOP16 and SCOOP24 (1 µM, grey). Each biological replicate (n=4 plants) is represented by four technical replicates. Significance was tested by performing a paired Wilcoxon rank-sum test.

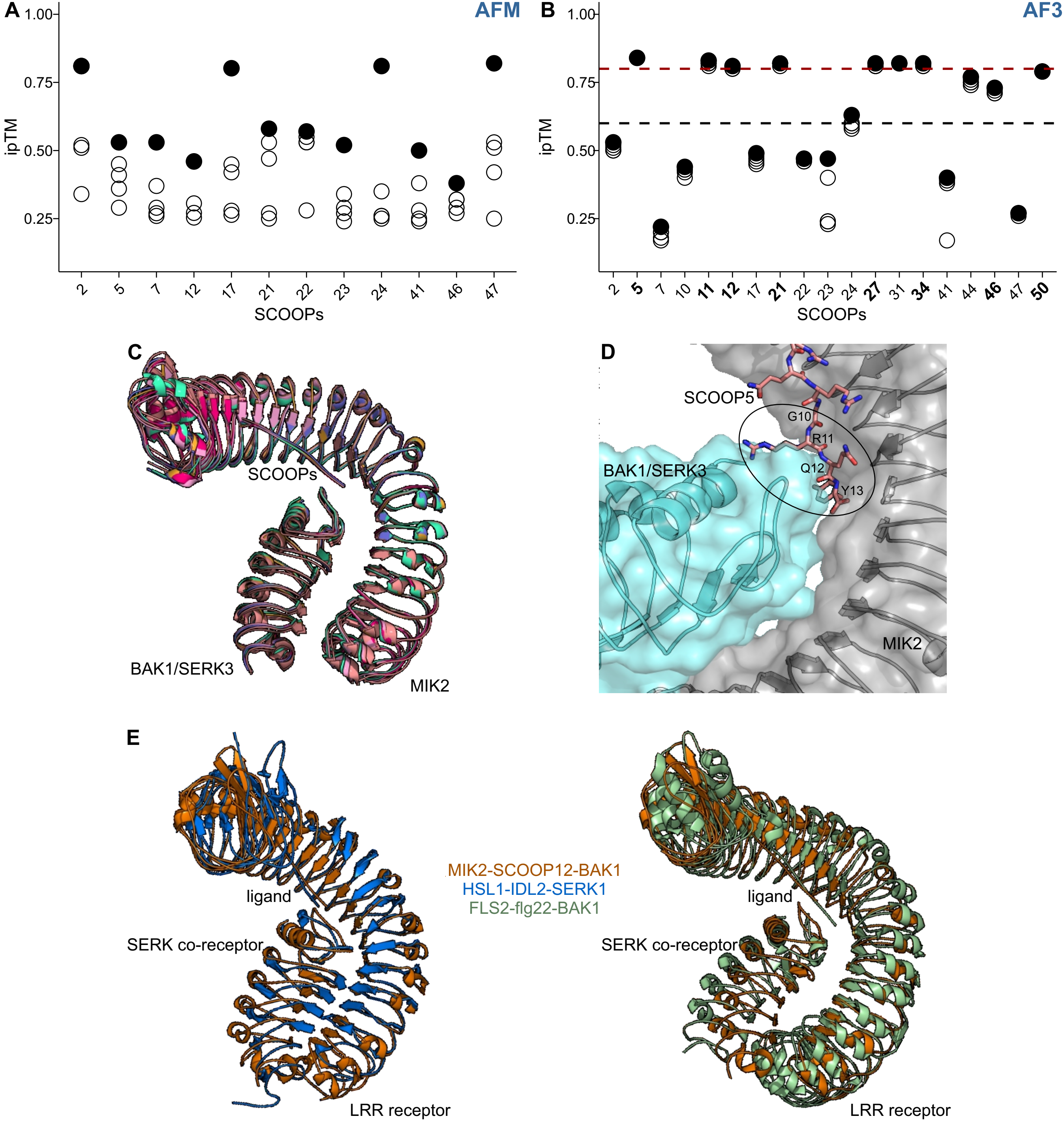


Fig. S4: **AlphaFold 3 predicts putative interaction interfaces of the tripartite MIK2-SCOOP-BAK1 complex**. **A-B)** AlphaFold-Multimer (AFM) and AlphafFold 3 (AF3) predict multiple high interface predicted Template Modelling (ipTM) scores for Arabidopsis SCOOPs in complex with the MIK2 receptor and BAK1 co-receptor. Eight AF3-predicted interactions of the tripartite complex have a consistent orientation of both the 13mer SCOOP and BAK1 and are highlighted in bold. The AF3 guidelines state that ipTM values higher than 0.8 represent confident high-quality predictions. ipTM values between 0.6 and 0.8 are within a gray zone where predictions could be correct or incorrect. The AF3 cut-offs are depicted with a red and black dotted line. **C)** Structural superposition of MIK2-SCOOPs-BAK1 predicted complexes with AF3. Cartoon representation of SCOOP12 (orange), SCOOP5 (salmon), SCOOP21 (light pink), SCOOP27 (violet), SCOOP11 (hotpink), SCOOP46 (cyan), SCOOP50 (pink). R.M.S.D of ~ 0.6-0.8 Å comparing between 598 and 620 pairs of corresponding Cα atoms between the different complexes. **D)** The co-receptor BAK1 in the predicted complex reads the encircled four last SCOOP amino acids with its N-terminal loop. Close-up view of the AF3 predicted MIK2-SCOOP5-BAK1 peptide binding pocket. MIK2 is depicted in cartoon and surface representation (grey), SCOOP5 is highlighted in sticks (salmon) and BAK1 is represented in cartoon and surface view (cyan). **E)** MIK2-SCOOP12-BAK1 Af3 predicted complex aligns closer to HSL1-IDLs-SERK1 complex. Structural superimposition of MIK2-SCOOP12-BAK1 (orange) and HSL1-IDL2-SERK1 (7OGQ) (blue) (R.M.S.D of 2 Å comparing 635 pairs of corresponding Cα atoms between the different complexes). Right, structural superposition MIK2-SCOOP12-BAK1 (orange) and FLS2-flg22-BAK1(green) (4MN8) (R.M.S.D of 3.6 Å comparing 652 pairs of corresponding Cα atoms between the different complexes).

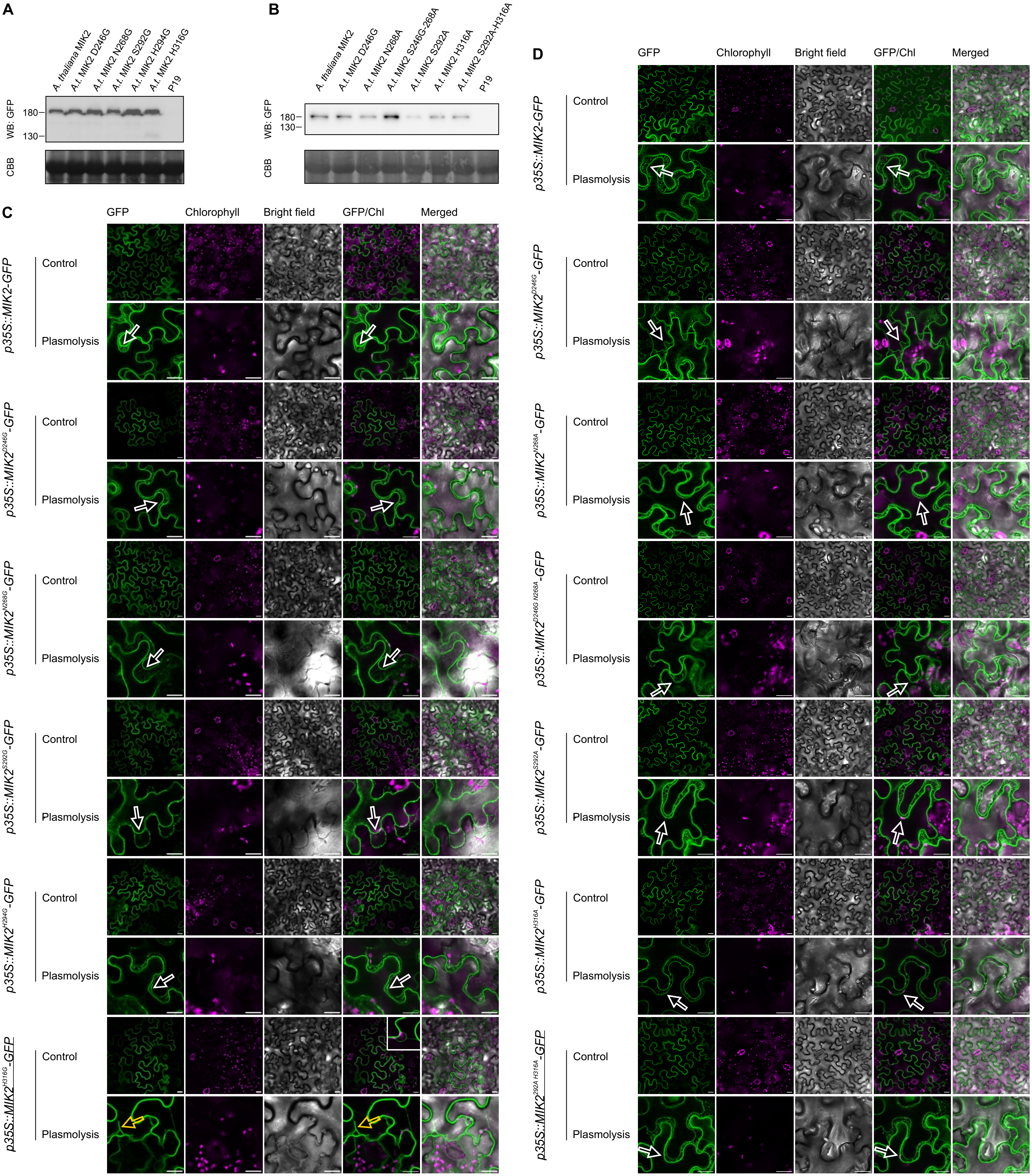


Fig. S5: **Confocal microscopy and western following the heterologous expression of MIK2 and MIK2 variants in *N. benthamiana*. A-B)** Western blot 72 h post-Agrobacterium infiltration. The western blot was probed with α-GFP (B-2) HRP as the receptor had a C-terminal GFP tag (top) and subsequently stained with CBB as a loading control (bottom). **C-D)** Confocal microscopy (GFP, Chlorophyll B and Bright Field) following Agrobacterium infiltration (72 h). All confocal microscopy images were identically modified, with small adjustments of brightness and contrast. The scale bar represents 20 μm. Plasmolysis was obtained by treatment with 0.6 M mannitol for 30 min. A repeat was performed and confirmed the depicted results. White arrows indicate correct localization at the plasma membrane, yellow arrows away from the plasma membrane.

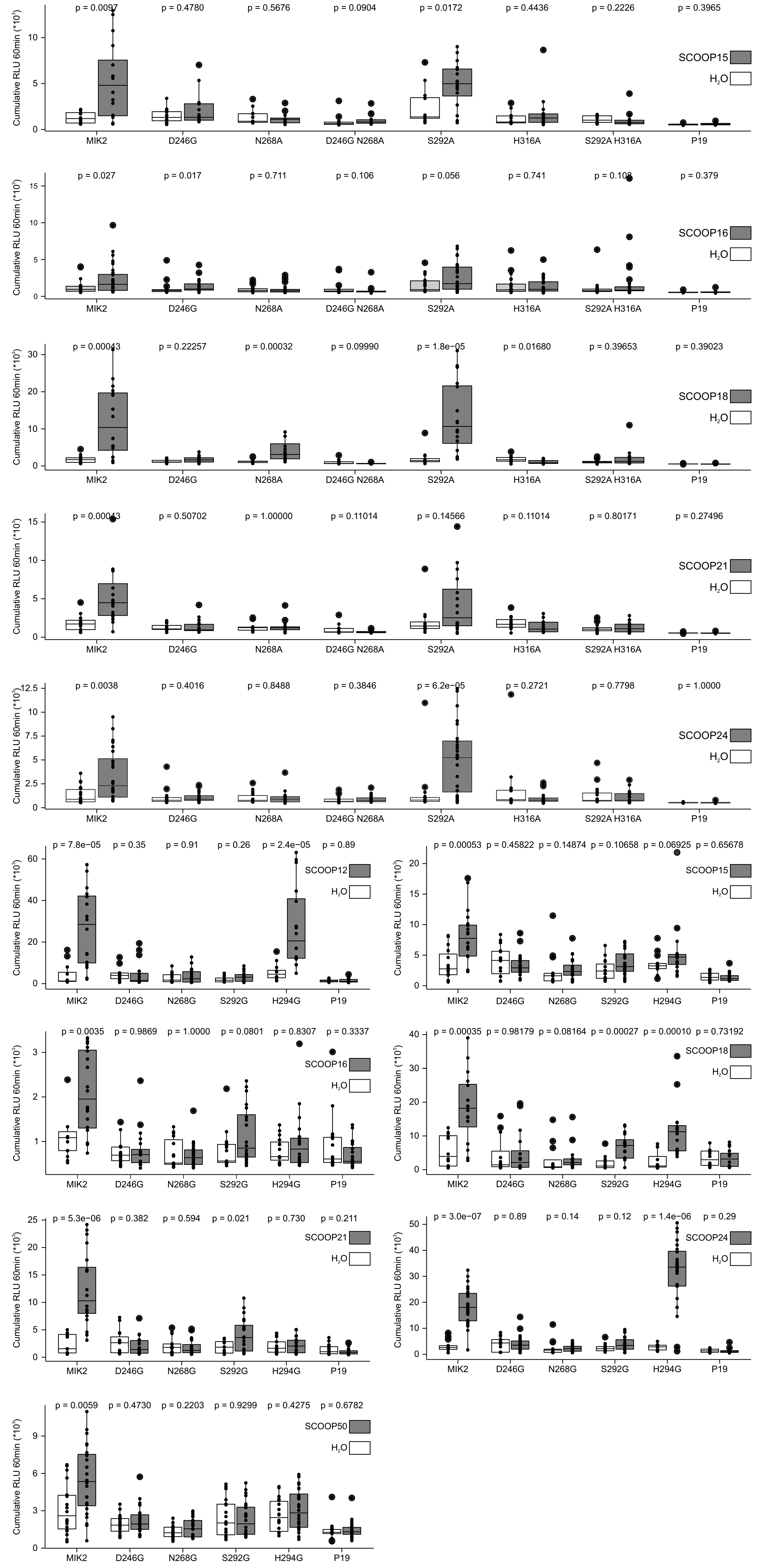


Fig. S6: **SCOOP-dependent reactive oxygen species (ROS) production following the heterologous expression of MIK2 and MIK2 variants in *N. benthamiana*.** At least four independent biological replicates were performed (n≥4 plants), with each biological replicate represented by four technical replicates. Cumulative RLUs representing ROS production are shown after treatment with $H_2O$ (white) or the SCOOP peptide indicated (1 µM, grey). Significance was tested by performing a paired Wilcoxon rank-sum test.

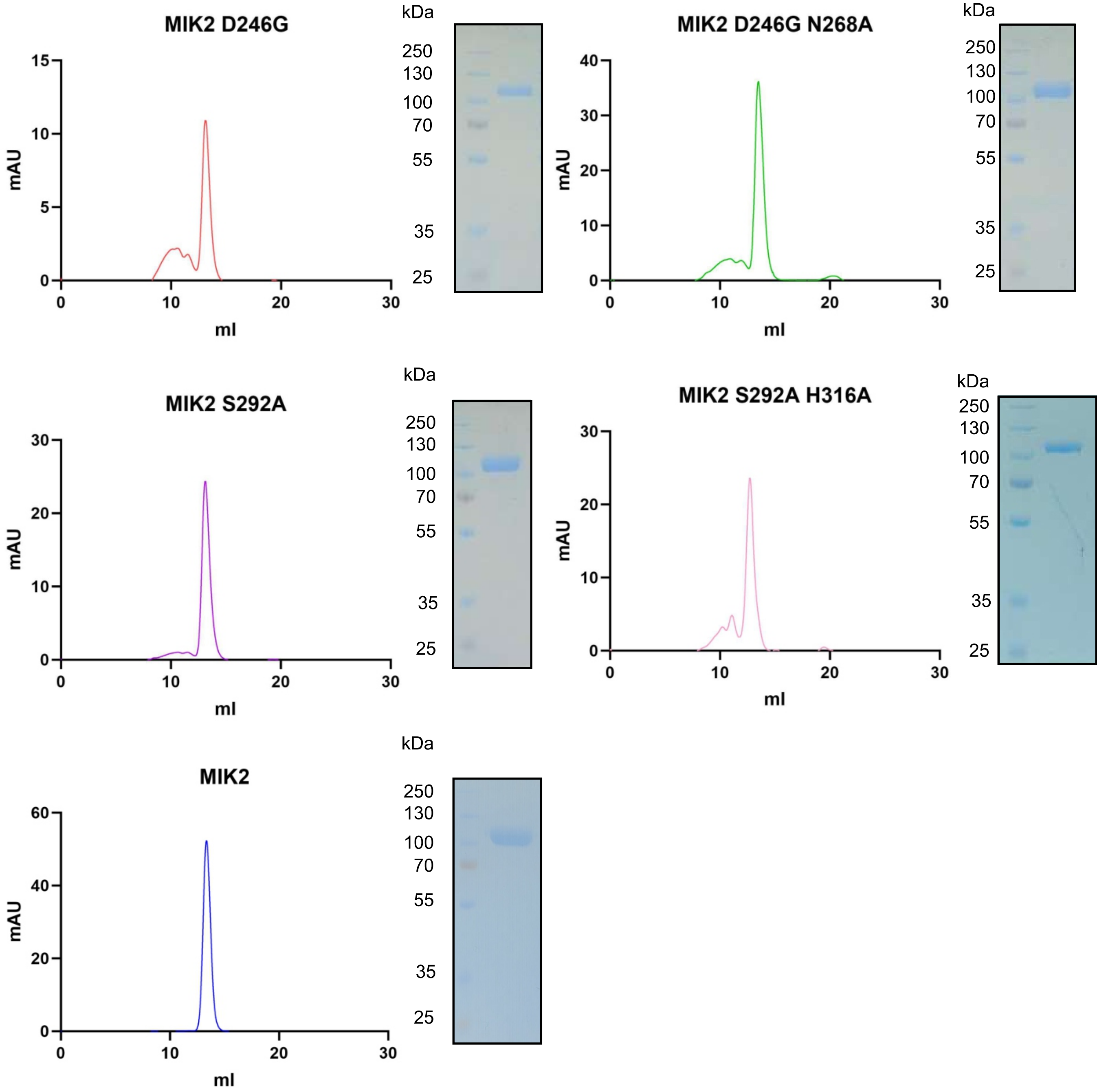


Fig S7. **Analytical size-exclusion chromatography experiments (SEC) of MIK2 pocket variants. SDS-PAGE of the proteins eluted are presented alongside.**

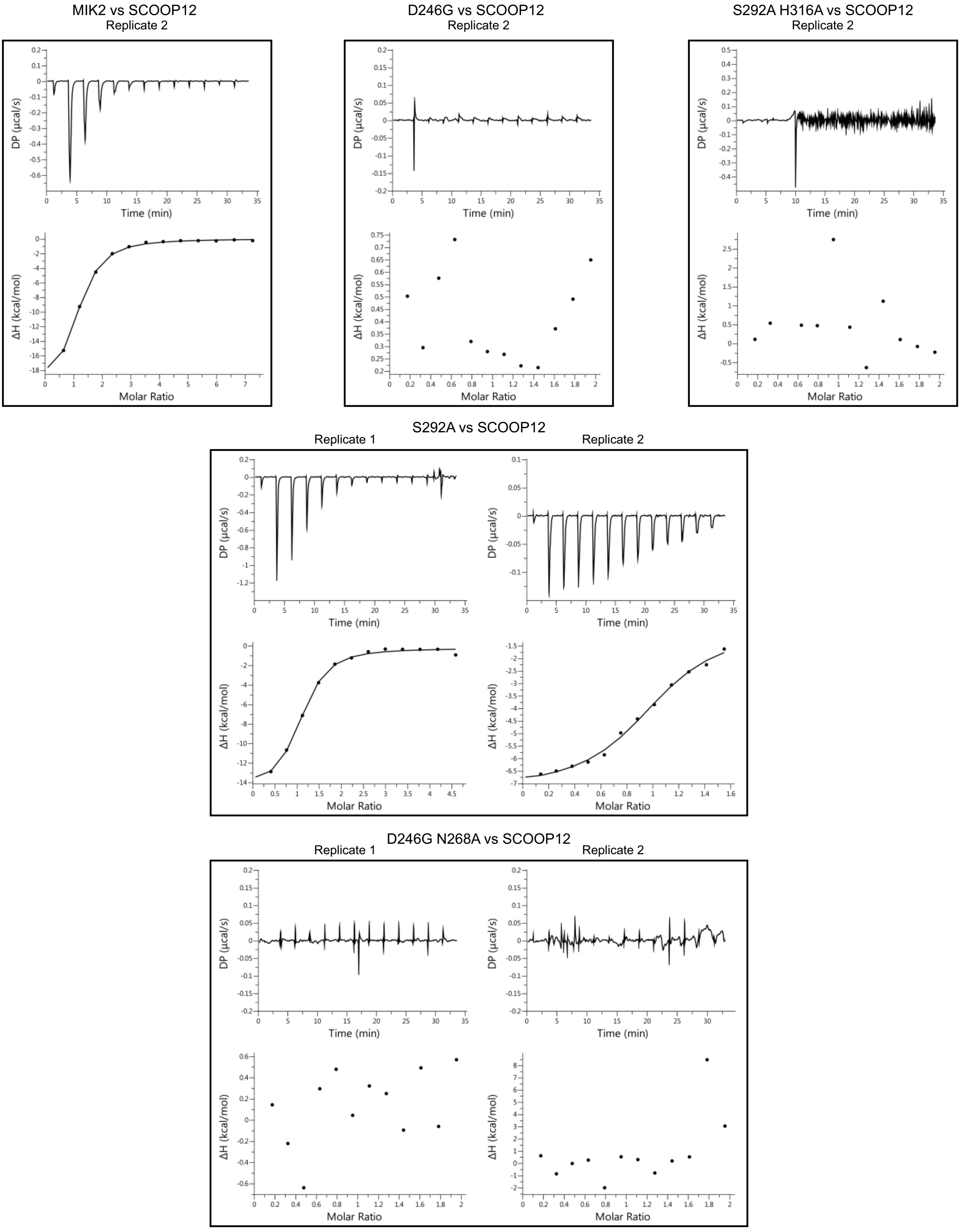


Fig S8. **ITC assays of MIK2 pocket variants and SCOOP12. ITC thermograms of the independent experiments performed for each mutant and analyzed in Fig. 4C.**

Table S1: **Primers used in this study.**

| Gene | Variant | Primer orientation | Sequence |
|---|---|---|---|
| *AT4G08850* | D246G | fw | GTCTAGgTAGGAACAACCTTACCGGTAAAATCCCTT |
| *AT4G08850* | D246G | rev | TTGTTCCTAcCTAGACATAGCTCTCTAAGGTTGGGTA |
| *AT4G08850* | N268G | fw | TCTTCTCggTATGTTTGAAAATCAGCTCTCTGGTG |
| *AT4G08850* | N268G | rev | AACATAccGAGAAGAGTTACATTCTTCAAATTCCCGAA |
| *AT4G08850* | S292G | fw | TACACTTgGTCTCCACACAAATAAGCTTACCGGT |
| *AT4G08850* | S292G | rev | GGAGACcAAGTGTATCTAAAGCGGTCATATTACCAA |
| *AT4G08850* | H294G | fw | GTCTCggCACAAATAAGCTTACCGGTCCAATAC |
| *AT4G08850* | H294G | rev | TTTGTGccGAGACTAAGTGTATCTAAAGCGGTCATA |
| *AT4G08850* | H316G | fw | CGTTCTTggTCTTTACCTGAATCAACTCAATGGTTC |
| *AT4G08850* | H316G | rev | TAAAGAccAAGAACGGCTAGGGTTTTGATGTTTCC |
| *AT4G08850* | N268A | fw | TCTTCTCgcTATGTTTGAAAATCAGCTCTCTGGTG |
| *AT4G08850* | N268A | rev | AACATAgcGAGAAGAGTTACATTCTTCAAATTCCCGAA |
| *AT4G08850* | S292A | fw | TACACTTgcTCTCCACACAAATAAGCTTACCGGT |
| *AT4G08850* | S292A | rev | GGAGAgcAAGTGTATCTAAAGCGGTCATATTACCAA |
| *AT4G08850* | H316A | fw | CGTTCTTgcTCTTTACCTGAATCAACTCAATGGTTC |
| *AT4G08850* | H316A | rev | TAAAGAgcAAGAACGGCTAGGGTTTTGATGTTTCC |

Dataset S1 (separate file): **Overview SCOOP mining.**

Dataset S2 (separate file): **Overview of mined assemblies for locus analyses, overview of Arabidopsis anchor genes *PROSCOOP* loci, and overview of contiguous *INR* loci (+ coordinates) and MIK2-orthologues.**

Dataset S3 (separate file): **Fasta file of the amino acid (AA) sequences of MIK2 homologues, putative MIK2 paralogues and outgroup included in the phylogenetic analysis.**

Dataset S4 (10.5281/zenodo.11615634): **Plasmid maps of constructs used in this study.**

Dataset S5 (10.5281/zenodo.11615634): **AFM and AF3 predicted structures (.pdb) and AFM confidence metrics (.pae).**

Dataset S6 (10.5281/zenodo.11615634): **Unedited files (.tiff) of co-IP and western blotting.**
